# Echinocystic Acid Bidesmoside Saponins from *Microglossa afzelii* O. Hoffm and Their Cytotoxic Activity against the CAL-27 Oral Squamous Carcinoma Cell Line

**DOI:** 10.3390/metabo12111018

**Published:** 2022-10-25

**Authors:** Billy Toussie Tchegnitegni, Tehmina Ahmed, Francis Tatong Ngouafong, Viviane Flore Kamlo Kamso, Rémy Bertrand Teponno, Bruno Ndjakou Lenta, Léon Azefack Tapondjou, Arslan Ali, Syed Ghulam Musharraf

**Affiliations:** 1 Department of Chemistry, Faculty of Science, University of Dschang, Dschang P.O. Box 67, Cameroon; 2 HEJ, Research Institute of Chemistry, International Center for Chemical and Biological Sciences, University of Karachi, Karachi 75270, Pakistan; 3 Panjwani Center for Molecular Medicine and Drug Research, International Center for Chemical and Biological Sciences, University of Karachi, Karachi 75270, Pakistan; 4 Department of Organic Chemistry, Faculty of Science, University of Yaoundé I, Yaoundé P.O. Box 812, Cameroon; 5 Department of Chemistry, Higher Teacher Training College, University of Yaoundé I, Yaoundé P.O. Box 47, Cameroon

**Keywords:** *Microglossa afzelii*, asteraceae, triterpenoid saponins, cytotoxic activity

## Abstract

This paper describes eight new triterpenoid saponins, including afzeliioside A (**1**), four acetylated afzeliiosides as pairs of inseparable regioisomers, called afzeliiosides B/C (**2/3**) and D/E (**4/5**), afzeliiosides F-H (**6–8**), and a known impatiprin C (**9**), which were isolated from the *n*-BuOH fraction of the liana of *Microglossa afzelii*. Their structures were established mainly by extensive spectroscopic analysis, including 1D and 2D NMR, HRFAB-MS, tandem ESI-MS/MS, and chemical methods, as well as a comparison of their spectral data with those of related compounds. All the isolates were screened for their cytotoxic activity against the CAL-27 oral squamous carcinoma cell line. Only compounds **4/5** (EC_50_ = 36.0 μg/mL (32.7 μM)) exhibited moderate cytotoxic activity. This work presents the first chemical and biological investigation of *Microglossa afzelii* and reports, for the first time, on the isolation of saponins in the genus *Microglossa*.

## 1. Introduction

Triterpenoid saponins, which are common in a large number of plant species, represent a class of secondary metabolites showing a remarkable structural variety, as well as notable biological activities [1]. Indeed, plants synthesize saponins as part of their normal program of growth and development and, in particular, as preformed chemical barriers functioning in defense mechanisms against insects, fungi, nematodes, and weeds [2]. The interest in saponins stems from their medicinal properties and their importance in the human and animal diet, since they have immunostimulant, hypocholesterolaemic, and anticarcinogenic properties and act as antifungal and antiviral agents [2].

An extremely rich chemical diversity characterizes the *Microglossa* species, as well as the other genera in the Asteraceae family. *Microglossa* (Asteraceae) is a genus of about 10 species, mainly distributed between South Africa, tropical Africa, and eastern Asia [3]. The plants of this genus are commonly used in folk medicine to treat several diseases. *M. pyrifolia* and *M. angolensis* are used in Kenya, Ghana and Madagascar to manage malaria, reduce the effects of malaria-related fevers, and treat gastro-intestinal disorders and microbial infections, and they are also used as abortifacients and antipyretics [4,5,6]. *M. afzelii* is used in the western region of Cameroon for the treatment of headaches and stomach pain [6].

Previous chemical investigations of the plants of this genus have identified diterpenoids, aurone glucosides, dihydrobenzofurans, polyacetylenes, flavonoids, and triterpenoids [3,5,7,8,9]. Furthermore, previous work performed on other genera of the Asteraceae family led to the isolation of several triterpene saponins with various biological activities, including cytotoxic activity against various cancer cell lines [10,11].

As part of our research on bioactive saponins from Cameroonian medicinal plants [12,13,14,15], we examined the *n*-BuOH fraction from the liana of *M. afzelii*, and this led to the isolation and structural analysis of eight new triterpenoid saponins, named afzeliiosides A–H (**1–8**), and one known compound (**9**), together with their cytotoxic activity against the CAL-27 oral squamous carcinoma cell line. This study reports on the isolation of the saponins from the plants of the genus *Microglossa* for the first time. The present work also provides evidence for future research on saponins, including not only those from *M. afzelii* but also from another species of this genus, which can be applied in further pharmacological and clinical applications.

## 2. Materials and Methods

### 2.1. General Methods

Optical rotations were measured using a JASCO P-2000 polarimeter. A JASCO 320-A spectrophotometer was used for the scanning of the IR spectra using KBr pellets. The 1D and 2D NMR spectra were run using Bruker Avance Neo NMR spectrometers operating at 500, 600, and 800 MHz for ^1^H and 125, 150, and 200 MHz for ^13^C. In this paper, all chemical shifts (*δ*) are given in ppm with reference to the residual solvent signal, and the coupling constants (*J*) are in Hz. FAB-MS and HRFAB-MS were recorded using a JEOL-600H-2 and JEOL HX 110 mass spectrometer for the low and high resolutions, respectively. ESIMSn was performed through FIA (flow injection analysis) using MeOH as the solvent (5 mL/min, nebulizer pressure 15 psi), with a drying gas flow of 4 L/min and drying gas temperature of 325 °C. The GC/MS was performed Agilent technology 7890A and 5975C, respectively. Column chromatography was performed using Sephadex LH-20 (eluted with MeOH) and silica gel 60 (0.040–0.063 mm, Merck) eluted with a mixture of EtOAc−MeOH−H_2_O. TLCs were carried out on precoated Kieselgel 60 F_254_ (Merck) plates developed with EtOAc−MeOH−H_2_O and on Kieselgel 60 RP-18 F254S (Merck KGaA 64271 Darmstadt, Germany) plates developed with MeOH–H_2_O mixtures. They were visualized by spraying with 5% methanolic H_2_SO_4_, followed by heating. Recycling preparative HPLC was performed using a Modal LC-908 pump connected to an ODS H-80 RP-C18 L1 series column (Japan Analytical Industries) and RI-5 and UV-310 detectors (Japan Analytical Industries). The flow rate was 3 mL/min, and 3 mL of solution was injected each time.

### 2.2. Plant Material

The liana of *Microglossa afzelii* O. Hoffm. was collected at Bangam, Bamendjou Sub-division, in the West Region of Cameroon in 2017. The plant material was identified by the botanist Mr. Nana Victor of the Cameroon National Herbarium, where a voucher specimen (no. 59821/HNC) was deposited.

### 2.3. Extraction and Isolation

The air-dried and powdered product of liana of *M. afzelii* (2.1 kg) was extracted with MeOH (12 L, 3 × 24 h) at room temperature, and the filtrate obtained was evaporated under vacuum conditions at 45 °C to yield the crude MeOH extract (135.7 g). Part of the extract (130 g) was suspended in distillated water (800 mL) and successively partitioned with *n*-hexane (1 L, 3 times), EtOAc (1 L, 3 times), and *n*-BuOH (1 L, 3 times) to give 26.7 g, 82.3 g, and 12.5 g of each fraction, respectively. An amount of 10 g of the *n*-BuOH extract was repeatedly subjected to Sephadex LH-20 column chromatography (CC) using MeOH as the eluent in order to separate the saponins from the polyphenolic compounds, sugars, and other classes of compounds. The enriched saponin fraction (4.3 g) was subjected to silica gel CC eluted with EtOAc−MeOH−H_2_O (95:5:2, *v*/*v*/*v*) to obtain three main subfractions, Fr1 (1.3 g), Fr2 (720 mg), and Fr3 (925.3 mg). Fr1 was submitted to CC eluted with the ternary system EtOAc−MeOH−H_2_O (18:2:1, *v*/*v*/*v*) to obtain two main fractions, Fr11 (233 mg) and Fr12 (112 mg). Fr11 was submitted to successive RP-18 recycling HPLC in isocratic conditions (MeOH−H_2_O, 3:2, *v*/*v*) to obtain **2/3** (17 mg, t_R_: 25.0 min). The Sephadex LH-20 CC of Fr12 eluted with MeOH afforded 9 (33 mg). Fr3 was submitted to CC eluted with the ternary system EtOAc−MeOH−H_2_O (17:2:1, *v*/*v*/*v*) to give three main fractions, Fr31 (157 mg), Fr32 (203 mg), and Fr33 (108 mg). Fr33 was submitted to successive RP-18 recycling HPLC in isocratic conditions (MeOH−H_2_O, 7:3, *v*/*v*) to obtain **4/5** (15 mg, t_R_: 28.0 min). The repeated RP-18 recycling HPLC of Fr32 in isocratic conditions (MeOH−H_2_O, 13:7, *v*/*v*) led to 1 (3.2 mg, t_R_: 15.0 min). The RP-18 recycling HPLC of Fr31 in isocratic conditions (MeOH−H_2_O, 1:1, *v*/*v*) afforded the mixtures **6–8** (10.3 mg, t_R_: 23.0 min).

*Afzeliioside A* (**1**): White powder; ${\left[\alpha \right]}_{D}^{25}$ +52.6 (*c* 0.5, MeOH); IR (KBr) 3377, 2905, 1728, 1468, 1037, 1022 cm^−1^; ^1^H and ^13^C NMR data, see Table 1; HRFAB−MS (negative mode) *m*/*z* = 1245.6211 [M−H]^−^ (Calcd for C_61_H_97_O_26_: 1245.6268), *m*/*z* = 1189.2 [M−H−57]^−^, *m*/*z* = 1013.2 [M−H−176−57]^−^, and *m*/*z* = 703.4 [M−H−134−134−148−134]^−^.

*Afzeliioside B/C* (**2/3**): White powder;$\;{\left[\alpha \right]}_{D}^{25}$ +11,4 (*c* 0.5, MeOH); IR (KBr) 3353, 2921, 1728, 1641, 1376, 1218, 1078 cm^−1^; ^1^H NMR (600 MHz, CD_3_OD) δ_H_ 1.04/1.05 (H-23), 0.84 × 2 (H-24), 0.95 × 2 (H-25), 0.77/0.78 (H-26), 1.35/1.36 (H-27), 0.87 × 2 (H-29), 0.94 × 2 (H-30), 5.29/5.31 (t, *J* = 3.7 Hz each, H-12), 5.47/5.41 (d, *J* = 6.0 Hz each, H-1‴), 5.22/5.23 (d, *J* = 1.8 Hz each, H-1⁗), 4.52/4.41 (d, *J* = 7.6 Hz each, H-1′′′′′) and 4.38 × 2 (d, *J* = 7.8 Hz, H-1′), 5.12 (dd, *J* = 1.8; 3.5 Hz, H-2⁗) and 5.04 (dd, *J* = 3.1; 9.2 Hz, H-3⁗); ^13^C NMR (150 MHz, CD_3_OD), see Table 2 and Table 3; HRFAB−MS (negative mode) *m*/*z* 1155.5981 [M−H]^−^ (Calcd for C_58_H_91_O_23_: 1155.5951).

*Afzeliioside D/E *(**4/5**): White powder;$\;{\left[\alpha \right]}_{D}^{25}$ +29.6 (*c* 0.5, MeOH); IR (KBr) 3296, 2927, 1729, 1562, 1414, 1254, 1050 cm^−1^; ^1^H NMR (600 MHz, CD_3_OD) δ_H_ 1.06 × 2 (H-23), 0.86 × 2 (H-24), 0.95 × 2 (H-25), 0.78/0.79 (H-26), 1.35/1.36 (H-27), 0.87 × 2 (H-29), 0.94/0.95 (H-30), 5.30/5.32 (H-12), 5.47/5.41 (d, *J* = 6.0 Hz each, H-1‴), 5.23/5.24 (d, *J* = 1.8 Hz each, H-1⁗), 4.53/4.42 (d, *J* = 7.6 Hz each, H-1′′′′′) and 4.38 × 2 (d, *J* = 7.8 Hz, H-1′), 5.12 (dd, *J* = 1.8; 3.5 Hz, H-2⁗) and 5.05 (dd, *J* = 3.1; 9.2 Hz, H-3⁗); ^13^C NMR (150 MHz, CD_3_OD), see Table 2 and Table 3; HRFAB−MS (negative mode) *m*/*z* 1099.5355 [M−H]^−^ (Calcd for C_54_H_83_O_23_: 1099.5325). ESI−MS^2^ *m*/*z* = 1099.2, *m*/*z* = 1057.4 [M−H−43]^−^, *m*/*z* = 967.5 [M−H−134]^−^, *m*/*z* = 779.2 [M−H−134−148−43]^−^ and *m*/*z* = 647.4 [M−H−134−148−43−134]^−^. MS^3^ *m*/*z* = 471.3 [647.4–176]^−^.

*Afzeliioside F-H* (**6–8**): White powder;$\;{\left[\alpha \right]}_{D}^{25}$ +7.0 (*c* 0.5, MeOH); IR (KBr) 3300, 2920, 1700, 1520, 1450, and 1050 cm^−1^. ^1^H NMR (800 MHz, CD_3_OD) δ_H_ 1.04 × 3 (H-23), 0.85 × 3 (H-24), 0.95 × 3 (H-25), 0.77–0.79 (H-26), 1.35–1.37 (H-27), 0.87 × 3 (H-29), 0.94 × 3 (H-30), 5.30–5.32 (H-12), 4.34 × 3 (d, *J* = 7.8 Hz, H-1′), 5.52/5.48/5.44 (d, *J* = 6.0 Hz each, H-1″), 5.33/5.23/5.15 (d, *J* = 1.8–32.0 Hz, H-1‴), and 5.12 (dd, *J* = 2.1 Hz, H-2‴); 4.87 (m, H-3‴) 4.93 (m, H-4‴) ^13^C NMR (200 MHz, CD_3_OD), see Table 4 and Table 5, HRFAB−MS (negative mode) *m/z* C_49_H_75_O_19_ (Calcd for C_49_H_75_O_19_: 967.4903), *m/z* = 925.4 [M−H−43]^−^, [M−H−148−43]^−^, [M−H−148−43−134]^−^.

### 2.4. Acid Hydrolysis and GC-MS Analysis of Saponins ***1–9***

Each saponin (2 mg) was refluxed with HCl (1 N, 2 mL) for 2 h. The hydrolyzed product was extracted with CH_2_Cl_2_. The residue obtained from the usual workup of the aqueous layer was derivatized by adding 100 μL methoxylamine hydrochloride in pyridine (20 mg·mL^−1^), vortexed and incubated at 60 °C for 45 min. Then, 200 μL of *N*,*O*-Bis(trimethylsilyl)trifluoroacetamide (BSTFA) was added and incubated at 60 °C for 45 min. The derivatized sample was centrifuged at 6000 rpm for 5 min at 25 °C to remove any solid debris. A GC-MS analysis was performed using a 7890A gas chromatograph (Agilent technologies, Santa Clara, CA, USA), equipped with an Agilent Technology GC autosampler 120 (PAL LHX-AG12) and coupled with an Agilent 7000 Triple Quad system (Agilent technologies, USA). An HP-5MS 30 m–250 mm (i.d.) fused-silica capillary column (Agilent J &W Scientific, Folsom, CA, USA), chemically bonded with a 5% diphenyl and 95% dimethylpolysiloxane cross-linked stationary phase (0.25 mm film thickness), was used. Separation was achieved using a temperature program of 80 °C for 2 min, which was then ramped at 5 °C min^−1^ to 300 °C and held for 1 min, at a constant flow of 1.0 mL·min^−1^. The MS interface and ion source were set to 280. The configuration was determined by comparing the retention time with the derivatives prepared in a similar manner from standard sugar (t_R_ D-xylose 27.2 min; t_R_ L-rhamnose 32.3 min; t_R_ D-glucuronic acid 29.2 min; t_R_ D-apiose 4.2 min) [16].

### 2.5. Culture Conditions

The human tongue squamous cell carcinoma cell line (CAL-27) (ATCC CRL-2095) is an adherent cell line that was provided by the cell culture biobank of PCMD, ICCBS, which was used in the study. The cells with early passage numbers (P10–P12) were detached using trypsin-EDTA (Thermo Fisher Scientific, Waltham, MA, USA) and grown in Dulbecco’s modified Eagle medium (DMEM) (Thermo Fisher Scientific, USA) supplemented with 10% fetal bovine serum (FBS) (Thermo Fisher Scientific, USA) and 1% antibiotic-antimycotic (Thermo Fisher Scientific, USA) in a humidified incubator supplemented with 5% CO_2_ at 37 °C. The stock solutions of the tested compounds were prepared at 250 μg/μL by solubilizing the compounds in DMSO. The stock solutions were diluted to 500 μg/mL in DMEM.

### 2.6. Cytotoxic Assay

The cytotoxic effect of the isolated saponins was evaluated using an MTT(3-(4,5-Dimethylthiazol-2-yl)-2,5-dipheynyltetrazolium bromide) in vitro cell viability assay. This assay enabled a general evaluation of the cytotoxic activity of the tested samples. Briefly, CAL-27 cells were grown in DMEM on 96-well plates up to 70% confluence. Each compound was preliminarily screened at the concentrations of 250 μg/mL and 125 μg/mL in triplicate. A compound that showed 50% inhibition or more at 125 ug/mL was further evaluated for the EC_50_ value. When evaluating the EC_50_ values of the active compounds, the compounds were tested within the concentration range from 250 μg/mL to 7.813 μg/mL, followed by incubation for 48 h at 37 °C. After incubation, the medium was removed, 200 μL of MTT reagent (500 μg/mL) was added, and the samples were incubated for 1.5 h. The MTT reagent was removed, and DMSO was added to dissolve the formazan crystals formed by a reduction in the MTT reagent. A micro-plate reader was used to measure the absorbance at 570 nm [17]. Each sample analysis was performed in triplicate. The EC_50_ was calculated by plotting a non-linear regression curve using GraphPad Prism 9 software.

## 3. Results

**Figure 1:**
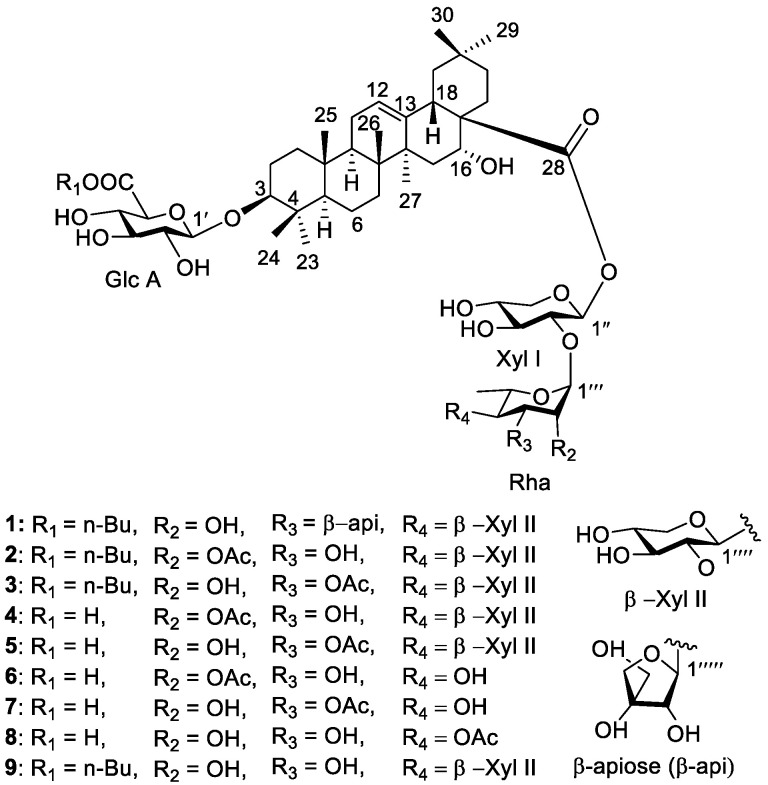
Structures of compounds **1–9** isolated from *M. afzelii*.

The *n*-BuOH fraction of the methanol extract of *M. afzelii* was subjected to Sephadex LH-20 column chromatography to obtain an enriched saponin fraction. Sephadex LH-20, open normal column chromatography, and the RP-18 HPLC of the enriched saponin fraction afforded one previously unreported triterpenoid saponin, named afzeliioside A (**1**), and three pairs of new ones, named afzeliiosides B/C (**2/3**) and afzeliiosides D/E (**4/5**), as well as afzeliiosides F-H (**6–8**), together with the known impatiprin C (**9**) [18] (Figure 1).

**Figure 2:**
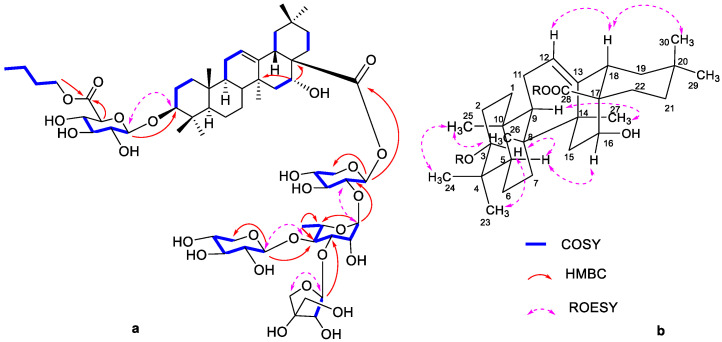
Key COSY, HMBC (**a**), and ROESY (**b**) correlations of **1**.

Compound **1** was isolated as a white powder from methanol, ${\left[\alpha \right]}_{D}^{25}$ +52.6 (*c* 0.5, MeOH). Its negative HRFAB−MS showed the deprotonated ion peak [M−H]^−^ at *m/z* 1245.6211, corresponding to the formula C_61_H_97_O_26_ (Calcd for C_61_H_97_O_26_: 1245.6268). The IR spectrum showed characteristic absorption bands for the hydroxyl groups (3377 cm^−1^) and ether groups (1037 cm^−1^), as well as a strong absorption due to the carbonyl groups (1728 cm^−1^) [15,19]. The analysis of the ^1^H and ^13^C NMR data (Table 1) indicated that **1** was a triterpene saponin containing five monosaccharides. The ^1^H NMR spectrum of **1** showed seven methyl signals at δ_H_ 1.04 (H-23), 0.84 (H-24), 0.95 (H-25), 0.77 (H-26), 1.36 (H-27), 0.86 (H-29), and 0.94 (H-30), and an olefinic proton at δ_H_ 5.31 (br t, *J* = 3.7 Hz, H-12) characteristic of olean-12-ene-type triterpenoids [20]. The ^13^C NMR spectrum showed 61 signals, among which 30 were assigned to a triterpenoid moiety and 27 to the saccharide moiety (see Table 1). The four additional signals were ascribable to an *n*-butyl (66.1 (CH_2_), 31.7 (CH_2_), 20.1 (CH_2_), and 14.0 (CH_3_)) unit, which was confirmed by the combined interpretation of the ^1^H−^1^H COSY, HSQC, and DEPT 135 spectra [18]. The NMR data of the aglycone moiety of **1** were in good agreement with those of echinocystic acid [18,21], except for the downfield shift of C-3 (δ_C_ 91.1) and for the upfield shift of C-28 (δ_C_ 177.2), suggesting that **1** was a bidesmosidic saponin [22,23]. The ^1^H NMR spectrum of **1** displayed the signals of five anomeric protons at δ_H_ 5.46 (d, *J* = 5.6 Hz, H-1‴), 5.16 (d, *J* = 1.8 Hz, H-1⁗), 4.62 (d, *J* = 7.8 Hz, H-1′′′′′), 5.26 (d, *J* = 3.8 Hz, H-1′′′′′′), and 4.38 (d, *J* = 7.8 Hz, H-1′), which correlated in the HSQC spectrum with the carbons at δ_C_ 95.4 (C-1‴), 101.1 (C-1⁗), 105.1 (C-1′′′′′), 111.9 (C-1′′′′′), and 107.0 (C-1′), respectively (Table 1). The ^1^H NMR spectrum also revealed the presence of one rhamnopyranosyle moiety amongst the sugars, and it was identified by the correlation between the methyl doublet at δ_H_ 1.26 (d, *J* = 6.2 Hz, H-6⁗) and the glycosidic proton at δ_H_ 3.76 (dd, *J* = 6.2; 9.5 Hz, H-5⁗). The acid hydrolysis of **1** afforded D-glucuronic acid, D-xylose, L-rhamnose, and D-apiose, which were identified by the comparison of the retention times of their trimethylsillyled derivatives with those of the authentic ones by GC-MS [16]. The *β*-configuration of the anomeric centers of glucuronic acid, xylose, and apiose, as well as the *α* of rhamnose, were determined based on the values of their coupling constants [23,24]. The complete assignments of the glycosidic protons and carbons (Table 1) were carried out through the analysis of the ^1^H−^1^H COSY, TOCSY−2D, and HSQC data. The correlations observed between H-3/H-23 and H-3/H-5 in the ROESY spectrum (Figure 2b) indicated the *β*-configuration of the oxygen at C-3 (δ_C_ 91.1). The location of the glucuronopyranosyl unit was deduced by the HMBC correlation of its anomeric proton (δ_H_ 4.38) with C-3. Furthermore, the upfield shift of C-6′ (170.9) suggested that it was esterified, and this was further confirmed by the HMBC correlation depicted between the H-1 (δ_H_ 4.18, t, *J* = 6.4 Hz) of *n*-butyl and this carbon. For the tetrasaccharide side chain at the C-28 position, the HMBC spectrum showed correlations of the H-1 of Xyl II (δ_H_ 4,62) with C-4 of Rha (δ_C_ 78.4), the H-1 of Api (δ_H_ 5.26) with the C-3 of Rha (δ_C_ 81.4), the H-1 of Rha (δ_H_ 5.16) with the C-2 of Xyl I (δ_C_ 76.5), and finally the H-1 of Xyl I (δ_H_ 5.46) with C-28 (δ_C_ 177.2) (Figure 2a). The negative-mode FAB mass spectrum showed some important fragment ion peaks at *m/z* = 1189.2 [M−H−57]^−^, *m/z* = 1013.2 [M−H−176−57]^−^, and *m/z* = 703.4 [M−H−134−134−148−134]^−^, attributed to the loss of *n*-butyl, *n*-butylglucuronopyranosyl, and the C-28 tetrasaccharide chain formed, of two xylopyranosyls, one apiofuranosyl, and rhamnopyranosyl moieties, respectively. On the basis of the above analysis, the structure of **1** was thus elucidated as 3-*O*-[(6-*O*-*n*-butyl)-*β*-*D*-glucuronopyranosyl]-echinocystic acid 28-*O*-[*β*-*D*-xylopyranosyl-(1→4)-[*β*-*D*-apiofuranosyl-(1→3)]-α-*L*-rhamnopyranosyl(1→2)-*β*-*D*-xylopyranoside (afzeliioside A).

Mixtures **2** and **3** were isolated as a white powder from methanol and appeared as a single spot on RP-18 TLC, ${\left[\alpha \right]}_{D}^{25}$ +11.4 (*c* 0.5, MeOH). The negative HRFAB−MS showed the deprotonated ion peak [M−H]^−^ at *m*/*z* 1155.5981, corresponding to the formula C_58_H_91_O_23_ (Calcd for C_58_H_91_O_23_: 1155.5951). The IR spectrum showed absorption bands at 3353, 1078, and 1728 cm^−1^, indicating the presence of hydroxyl groups, ether linkage, and carbonyl groups, respectively [15,19,25]. It was shown to be a mixture of the two monoacetylated olean-12-ene saponins **2** and **3**, in the proportions of 1:1, based on the interpretation of the NMR data. The ^1^H NMR spectrum of the aglycone portion of **2/3** showed seven methyl signals as pair of protons at δ_H_ 1.04/1.05 (H-23), 0.84 × 2 (H-24), 0.95 × 2 (H-25), 0.77/0.78 (H-26), 1.35/1.36 (H-27), 0.87 × 2 (H-29), and 0.94 × 2 (H-30), and characteristic olefinic protons at δ_H_ 5.29/5.31 (t each, *J* = 3.7 Hz, H-12). The ^13^C NMR spectrum showed 58 signals, among which 30 were assigned to a triterpenoid moiety, 22 to the saccharide portion (see Table 1 and Table 2), and 2 to an acetyl group with the methyl signals at δ_C_ 20.9/21.3 (s, MeCO), as well as the signals of ester carbonyls at δ_C_ 172.2/172.5 (MeCO). The four additional signals were ascribable to an *n*-butyl (66.1 × 2 (CH_2_), 31.7 × 2 (CH_2_), 20.2 × 2 (CH_2_) and 14.0 × 2 (CH_3_)) unit based on the interpretation of the ^1^H−^1^H COSY, HSQC, and DEPT 135 spectra [18]. The comparison of the NMR data of the aglycone moiety of **2/3** with that of **1** showed that they are superimposable and indicated that **2/3** are also echinocystic acid glycosides [18,25]. Furthermore, the chemical shifts of C-3 (δ_C_ 91.1 × 2) and C-28 (δ_C_ 177.0/177.1) suggested that **2/3** were also bidesmosidic saponins [22,23]. The ^1^H NMR spectrum of **2/3** displayed the anomeric protons of four sugar units at δ_H_ 5.47/5.41 (d, *J* = 6.0 Hz each, H-1‴), 5.22/5.23 (d, *J* = 1.8 Hz each, H-1⁗), 4.52/4.41 (d, *J* = 7.6 Hz each, H-1′′′′′), and 4.38 × 2 (d, *J* = 7.8 Hz, H-1′), which correlated in the HSQC with four anomeric carbons at δ_C_ 95.5/95.4 (C-1‴), 98.4/101.0 (C-1⁗), 106.5/105.6 (C-1′′′′′), and 107.0 × 2 (C-1′), respectively (Table 2), confirming that each constituent of the mixture **2/3** contained four sugar units. The acid hydrolysis of **2/3** afforded glucuronic acid, xylose, and rhamnose, which were identified by the comparison of their retention times with those of the authentic trimethylsillyled derivatives by GC-MS [16,19]. Furthermore, the D-configuration of the glucuronic acid, xylose, and L of rhamnose were also determined by the GC-MS analysis of their trimethylsillylated derivatives. The *β*-configuration of the anomeric centers of the glucuronic acid and xylose and *α* of rhamnose were determined based on the values of their coupling constants [23,24]. An extensive analysis of the ^1^H−^1^H COSY spectrum allowed us to assign the resonances observed in a lower field at δ_H_ 5.12 (dd, *J* = 1.8; 3.5 Hz) and 5.04 (dd, *J* = 3.1; 9.2 Hz) to H-2⁗ and H-3⁗ of the two rhamnopyranosyl units, respectively, and suggested that they were acetylated. This was further confirmed by the HMBC correlation depicted between H-2⁗ (δ_H_ 5.12) and H-3⁗ (δ_H_ 5.04) and the acetyl carbonyl carbons at δ_C_ 172.2 and 172.5, respectively, indicating that **2/3** were a mixture of 2-OAc and 3-OAc-rhamnopyranosyl regioisomers. The location of the glucuronopyranosyl unit at C-3 (δ_C_ 91.1 × 2) was deduced based on the HMBC correlation of its anomeric proton (δ_H_ 4.38 × 2) with C-3. The correlation of H-1 (δ_H_ 4.18, t, *J* = 6.4 Hz) of the *n*-butyl w C-6 (170.9 × 2) of the glucuronopyranosyl unit allowed us to locate the *n*-butyl moiety in this position. The linkage of the trisaccharide side chain in the C-28 position of the aglycone was evidenced by the HMBC correlation of the H-1 of Xyl II (δ_H_ 4.52/4.41) with the C-4 of Rha (δ_C_ 83.2/77.3), the H-1 of Rha (δ_H_ 5.23/5.22) with the C-2 of Xyl I (δ_C_ 76.0/76.6), and the H-1 of Xyl I (δ_H_ 5.47/5.41) with the C-28 (δ_C_ 177.0/177.1). The negative-mode FAB mass spectrum showed some important fragment ion peaks at *m/z* = 1113.1 [M−H−43]^−^, *m/z* = 923.0 [M−H−176−57]^−^, *m/z* = 703.4 [M−H−134−134−148−43]^−^, and *m/z* = 471.1 [M−H−2×134−148−43−176−57], attributed to the loss of an acetyl, a *n*-butylglucuronopyranosyl, a C-28 triglycosidic chain formed of two xylopyranosyls, one rhamnopyranosyl, and acetyl moieties, and the loss of the C-3,28 glycosidic chains, respectively. On the basis of the above analysis, the structure of **2/3** was thus elucidated as a mixture of 3-*O*-[(6-*O*-*n*-butyl)-*β*-*D*-glucuronopyranosyl]-echinocystic acid 28-*O*-[*β*-*D*-xylopyranosyl]-(1→4)-2-*O*-acetyl-α-*L*-rhamnopyranosyl-(1→2)-*β*-*D*-xylopyranoside (afzeliioside B) and 3-*O*-[(6-*O*-*n*-butyl)-*β*-*D*-glucuronopyranosyl]-echinocystic acid 28-*O*-[*β*-*D*-xylopyranosyl]-(1→4)-3-*O*-acetyl-α-*L*-rhamnopyranosyl-(1→2)-*β*-*D*-xylopyranoside (afzeliioside C).

**Scheme 1:**
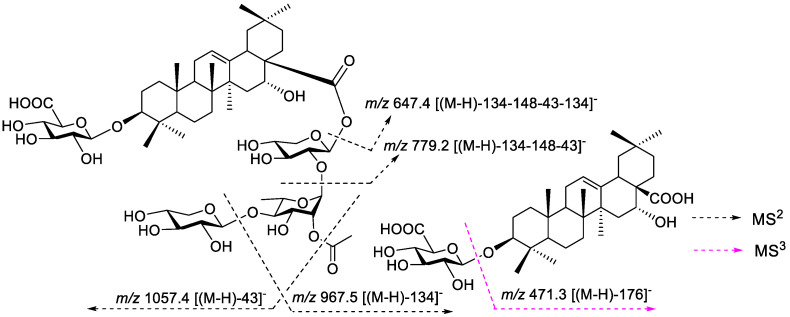
Ion peak *m/z* 1099.2 and ESI-MS^3^ of sub-ion peak *m/z* 647.4 of **4/5**.

Mixtures **4** and **5** were isolated as a white powder from methanol and appeared as a single spot on RP-18 TLC, ${\left[\alpha \right]}_{D}^{25}$ +29.6 (*c* 0.5, MeOH). The negative HRFAB−MS showed the deprotonated ion peak [M−H]^−^ at *m*/*z* 1099.5355, corresponding to the formula C_58_H_91_O_23_ (Calcd for C_54_H_83_O_23_: 1099.5325), with 56 mass units, which was less than **2/3**. The IR spectrum showed characteristic absorption bands at 3296, 1050, and 1729 cm^−1^, indicating the presence of the hydroxyl groups, ether linkage, and carbonyl groups, respectively [15,19,25]. The ^1^H NMR spectrum displayed the signals of seven methyl groups as pairs of protons at δ_H_ 1.06 × 2 (H-23), 0.86 × 2 (H-24), 0.95 × 2 (H-25), 0.78/0.79 (H-26), 1.35/1.36 (H-27), 0.87 × 2 (H-29), and 0.94/0.95 (H-30), together with the olefinic proton signal at δ_H_ 5.30/5.32 (t each, *J* = 3.5 Hz, H-12). The ^13^C NMR spectrum of **4/5** (1:1) displayed characteristic signals of monoacetylated bidesmosidic triterpenoid saponins with echinocystic acid as an aglycone at δ_C_ 90.8/90.9 (C-3), 123.6 × 2 (C-12), 144.6/144.7 (C-13), 74.6 × 2 (C-16), 177.0 × 2 (C-28), 21.3/21.4 (CH_3_CO), and 172.0/172.1 (CH_3_CO) [18]. The signals at δ_C_ 95.5/95.4 (C-1‴), 98.4/101.1 (C-1⁗), 106.7/105.5 (C-1′′′′′), and 106.5/106.9 (C-1′) indicated that **4/5** possessed four sugar units. The duplication of the ^1^H and ^13^C signals indicated that **4/5** also constituted a mixture. The GC-MS analysis of the chiral derivatives of sugars from the acid hydrolysate of **4/5** afforded D-glucuronic acid, D-xylose, and L-rhamnose [16]. The comparison of the NMR spectra of **4/5** with those of **2/3** showed that they were almost superimposable. However, the extensive analysis of the ^13^C NMR of **4/5** showed the disappearance of the signals attributable to an *n*-butyl unit, and this was further confirmed by the mass difference of 56 units between the two compounds, supporting the lack of a butyl unit. Tandem mass spectrometry (MS^n^), which utilizes the collision-induced dissociation (CID) of target ions, was used to confirm the sequences of the sugar chains. The negative ESI−MS^2^ analysis of the parent peak at *m*/*z* = 1099.2 gave fragments at *m*/*z* = 1057.4 [M−H−43]^−^, *m*/*z* = 967.5 [M−H−134]^−^, *m*/*z* = 779.2 [M−H−134−148−43]^−^, and *m*/*z* = 647.4 [M−H−134−148−43−134]^−^, attributed to the loss of acetyl, terminal xylopyranosyl, acetyl rhamnopyranosyl, and the C-28 triglycosidic chain, formed of two xylopyranosyl units and acetyl rhamnopyranosyl, respectively. The MS^3^ of *m*/*z* = 647.4 gave an ion peak at *m*/*z* = 471.3 [647.4–H–176]^−^, indicating the loss of a glucuronopyranosyl unit (Scheme 1). Therefore, the structure of **4/5** was elucidated as a mixture of 3-*O*-*β*-*D*-glucuronopyranosylechinocystic acid 28-*O*-[*β*-*D*-xylopyranosyl]-(1→4)-2-*O*-acetyl-α-*L*-rhamnopyranosyl-(1→2)-*β*-*D*-xylopyranoside (afzeliioside D) and 3-*O*-*β*-*D*-glucuronopyranosylechinocystic acid 28-*O*-[*β*-*D*-xylopyranosyl]-(1→4)-3-*O*-acetyl-α-*L*-rhamnopyranosyl-(1→2)-*β*-*D*-xylopyranoside (afzeliioside E).

Mixtures **6–8** were obtained as a white powder from methanol and appeared as a single spot on RP-18 TLC, ${\left[\alpha \right]}_{D}^{25}$ +7.0 (*c* 0.5, MeOH) with the same molecular formula, deduced from the negative HRFAB−MS, which showed the deprotonated ion peak [M−H]^−^ at *m/z* 967.4951, corresponding to the formula C_49_H_75_O_19_ (Calcd for C_49_H_75_O_19_: 967.4903) and suggesting the presence of isomers. We observed 134 mass units, which marked a difference from **4/5** and indicated the loss of one pentose unit. The IR spectrum showed characteristic absorption bands at 3300, 1050, and 1700 cm^−1^, indicating the presence of the hydroxyl groups, ether linkage, and carbonyl groups, respectively [15,19,25].

The relative intensity of the proton signals and their combination with the ^13^C NMR spectra suggested that **6–8**, in 1:2:1 proportions, are also triterpenoid saponins with echinocystic as an aglycone. This was shown by the characteristic signals at δ_C_ 2 × 90.8/90.7 (C-3), 2 × 123.6/123.7 (C-12), 145.5/145.6/145.7 (C-13), 74.6/74.7/74.9 (C-16), and 177.0/177.1/177.2 (C-28) [18]. The signals which appeared as a triplicate at δ_C_ 20.5/172.4, 21.1/172.7, and 21.2/172.6 suggested that, as in the case of **4/5**, each constituent of the sample was monoacetylated. The extensive analysis of the sugar moiety revealed that each constituent contained three sugar units, and this was confirmed by the presence of three couples of anomeric carbons and protons at δ_C/H_ 3 × 106.4/4.34 (C-1′/H-1′), 95.5/5.52, 95.3/5.48 and 95.4/5.44 (C-1″/H-1″), and 101.5/5.33, 101.5/5.23, and 101.6/5.15 (C-1‴/H-1‴). The GC-MS analysis of the chiral derivatives of sugars from the acid hydrolysate of the sample afforded D-glucuronopyranosyl acid, D-xylopyranose, and L-rhamnopyranopyranosse [16], and the configurations of the anomeric centers were determined based on the values of their coupling constants [23,24]. An extensive analysis of the ^1^H−^1^H COSY and HSQC spectra allowed us to assign the resonances observed in a lower field at δ_C/H_ 73.9/5.12 (C-2‴/H-2‴), 75.8/4.87 (C-3‴/H-3‴), and 75.4/4.93 (C-4‴/H-4‴) to H-2‴, H-3‴, and H-4‴ of the three rhamnopyranosyl units, respectively, and suggested that they were acetylated. This was further confirmed by the HMBC correlation between H-2‴ (δ_H_ 5.12), H-3‴ (δ_H_ 4.87), and H-4‴ (δ_H_ 4.93) and the acetyl carbonyl carbons at δ_C_ 172.4, 172.7, and 172.6, respectively, indicating a mixture of 2-OAc, 3-OAc, and 4-OAc-rhamnopyranosyl regioisomers. The HMBC correlation between the anomeric protons at δ_H_ 3 × 4.34 (H-1′) and carbons at δ_C_ 2 × 90.8/90.7 (C-3) allowed us to link the glucuronopyranosyl unit at C-3. In addition, the linkage of the disaccharide side chain in the C-28 position of the aglycone was evidenced by the HMBC correlation of the H-1 of Rha (δ_H_ 5.33/5.23/5.15) with the C-2 of Xyl (δ_C_ 2 × 76.9/76.8) and the H-1 of Xyl (δ_H_ 5.52/5.48/5.44) with C-28 (δ_C_ 177.0/177.1/177.2). The negative-mode FAB mass spectrum showed some important fragment ion peaks at *m/z* = 925.4 [M−H−43]^−^, *m/z* = 779.3 [M−H−148−43]^−^, and *m/z* = 647.3 [M−H−148−43−134]^−^, attributed to the loss of acetyl, one monoacetylrhamnopyranosyl, and the disaccharide side chain formed od one acetyl, one rhamnopyranosyl, and one xylopyranosyl moiety, respectively. Based on the above information, the sample was elucidated as a mixture of three compounds, namely 3-*O*-*β*-*D*-glucuronopyranosylechinocystic acid 28-*O*-[2-*O*-acetyl-α-*L*-rhamnopyranosyl-(1→2)-*β*-*D*-xylopyranoside (afzeliioside F), 3-*O*-*β*-*D*-glucuronopyranosylechinocystic acid 28-*O*-[3-*O*-acetyl-α-*L*-rhamnopyranosyl-(1→2)-*β*-*D*-xylopyranoside (afzeliioside G), and 3-*O*-*β*-*D*-glucuronopyranosylechinocystic acid 28-*O*-[4-*O*-acetyl-α-*L*-rhamnopyranosyl-(1→2)-*β*-*D*-xylopyranoside (afzeliioside H).

**Figure 3:**
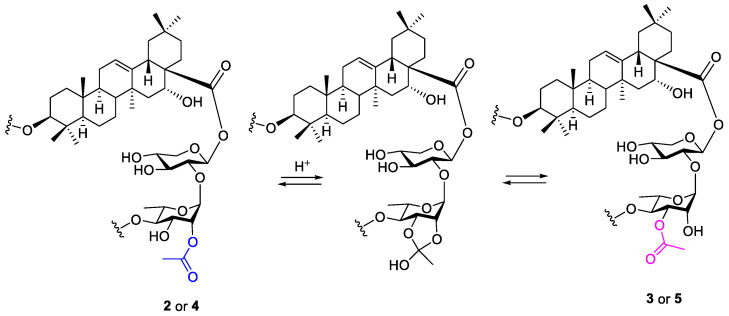
Possible mechanism of acetyl group transfer from **2** or **4** to **3** or **5** in [26].

Compounds **2/3**, **4/5**, and **6–8** are acetylated saponins, which are well known to be a typically unstable class of secondary metabolites that are extensively distributed in many species [15,19,26]. Their stability depends on the different solvents and separation materials used for their purification [26]. Due to the low activation energy of the acetyl migration reaction, it may be difficult to obtain the pure acetyl saponin [19,26]. According to Zeng et al. (2015) [26], the acetyl transfer reaction in saponins is faster in a mixture of solvents which contains water than in other solvents, and when comparing the normal/reverse silica gels, the reaction of acetyl migration almost did not occur during the process of purification by macroporous resin. Therefore, it can be assumed that compound **3** (or **5**) may have resulted from compound **2** (or **4**), and vice versa, due to the migration of the acetyl group from the 2 to the 3 position (vice versa) of the rhamnopyranosyl moiety, as shown by Zeng et al. (2015) [26] (Figure 3), thus resulting in the two inseparable mixtures of **2/3** and **4/5**. Regarding the mixtures of **6–8**, their appearance as three monoacetylated regioisomers is not surprising and could be due to the presence of three possible positions of acetyl migration in the rhamnopyranosyl unit, according to Zeng et al. (2015), compared to **2/3** and **4/5**, in which we observed two. We could then recommend using a macroporous resin as a stationary phase and avoiding the use of water as a solvent for the phytochemical investigation of acetyl-saponin-rich plants.

**Figure 4:**
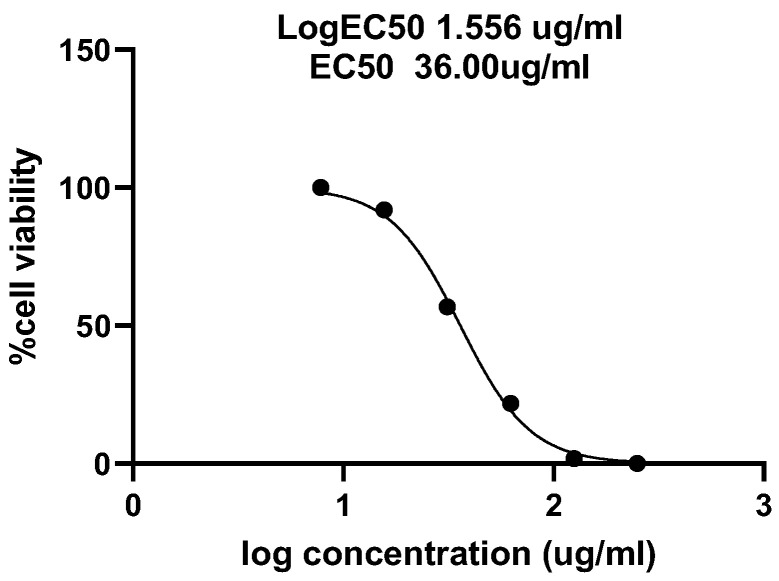
Cytotoxic effect of **4/5** against the oral squamous carcinoma cell line after 48 h of incubation.

Several studies have demonstrated that the polyhydroxylated triterpenes isolated from members of the Asteraceae family exhibit potent anti-inflammatory effects, as well as antitumor and cytotoxic activities [23,24,25]. Moreover, the work conducted by Wang et al. (2006) [27] reported that an oleanane triterpenoid possessing both 3-β and 6-α-hydroxyl groups exhibited anti-tumor activity against a diverse panel of tumor cell lines [27]. Taking into account the unusual structural features of *Microglossa* saponins, in order to determine their possible functional roles as cancer preventives, the isolated saponins (**1−9**) were screened for their cytotoxic activity against the CAL-27 oral squamous carcinoma cell line. The viability of the CAL-27 cell line incubated with the isolates was assessed using an MTT (3-(4,5-dimethyl thiazolyl-2)-2,5-diphenyltetrazolium bromide) assay [17]. Only **4/5** showed a moderate activity, with an EC_50_ = 36 μg/mL (32.7 μM) (Figure 4), while the other saponins were found to be inactive. (See Supplementary Figure S51) It is well known that the linkage of the sugar moieties, as well as their number and nature, are very important for the cytotoxicity of triterpenoid saponins [25]. The previous results on the cytotoxicity of triterpenoid saponins against various cancer cell lines showed that the C-3,28-bidesmosides were noncytotoxic, in most cases, compared to the C-3 ones [25,28,29]. Additionally, the work conducted by Lee et al. (2002) [25] revealed that the linkages of sugars at the C-28 of echinocystic acid are essential for the non-cytotoxicity trait. Furthermore, the main differences between **4/5**, which showed a moderate activity, and the other saponins are the lack of the *n*-butyl unit at the C-6 of the glucuronopyranosyl and the presence of one xylopyranosyl, which consequently, revealed that the carboxyl group and xylopyranosyl unit are essential for the observed activity.

## 4. Discussion

The present study reports, the first time, the isolation of triterpene saponins from the *Microglossa* genus. All the isolated compounds have echinocystic acid as an aglycone. The isolation of the triterpene saponins, as a major class of compounds from the *n*-BuOH fraction of this plant, was not surprising, since some triterpenoids from the less polar fraction of *Microglossa pyrifolia* have been reported [7]. This indicates that the chemical investigation of the polar fraction of this plant could yield related saponins. Moreover, previous works performed on other genera of the Asteraceae family led to the isolation of several triterpene-type saponins with various biological activities. Such is the case of *Silphium radula*, which afforded nine new saponins with cytotoxic activity against the breast cancer cell line [10]. From *Lactuca scariola*, one new saponin with antibacterial activity was isolated [30]. Furthermore, from *Viguiera hypargyrea*, four saponins were obtained, among which two new derivatives were isolated, but they exhibited no significant antiplasmodial or antibacterial activities [31]. Additionally, from *Atractylis flava*, three new saponins were isolated [32], while five saponins with one new saponin were isolated from *Calendula arvensis* [32], and from *Aster sedifolius* three new saponins were isolated [33]. Since all the isolated saponins from these genera are triterpenic with oleanane-based skeletons, it is noteworthy that this class has a broad spectrum of biological activities, including hepatoprotective, molluscicidal, anti-inflammatory, anti-tumor, immunomodulatory, anti-Alzheimer, hemolytic, and anti-allergic activities [11]. These chemical findings confirm the botanical identification of *M. afzellii* and further indicate its close relationship with other species of this genus. Additionally, echinocystic acid saponin derivatives, which constitute the main class of secondary metabolites isolated from the *n*-BuOH fraction of this plant, can thus be considered as chemotaxonomic markers of this species.

## 5. Conclusions

The chemical study of the *n*-BuOH fraction of *Microglossa afzelii* led to the isolation of eight new echinocystic-type triterpenoid saponins, trivially named afzeliiosides A−H (**1–8**), together with the known impatiprin C (**9**). Only the mixture of **4/5** displayed a moderate cytotoxic activity against the selected cancer cell lines. To the best of our knowledge, this study constitutes the first isolation of triterpene saponins from the *Microglossa* genus. The results obtained in this work indicated that echinocystic-type triterpenoid saponins represent one of the main classes of secondary metabolites derived from this plant and can thus be considered as its chemophenetic marker. Although the isolated saponins exhibited no significant cytotoxic activity, it would be interesting to re-isolate these saponins in large amounts and perform the cytotoxicity analysis using other cancer cell lines.

## Figures and Tables

**Table 1:** ^1^H and ^13^C NMR data of compound **1** (600 and 150 MHz, CD_3_OD).

Position	1				
	δ_C_	δ_H_		δ_C_	δ_H_
1	39.9	0.97 1.61	3-*O*-Glc A		
2	27.1	1.67 1.81	1′	107.0	4.38, d (7.8)
3	91.1	3.15	2′	75.3	3.22, dd (7.8; 2)
4	40.2		3′	77.9	3.84
5	57.1	0.76, d (3.7)	4′	73.0	3.52
6	19.3	1.60	5′	76.7	3.78, d (9.8)
7	34.3	1.38 1.52	6′	170.9	
8	40.8		Ester		
9	48.1	1.62	1″	66.1	4.18, t (6.4)
10	37.9		2″	31.7	1.64
11	24.5	1.88, d (5.8)	3″	20.1	1.42
12	123.5	5.31, br t (3.7)	4″	14.0	0.95
13	144.7		28-*O*-Xyl I		
14	42.7		1‴	95.4	5.46, d (5.6)
15	36.5	1.41, d (4.1) 1.74, dd (4.0; 15.0)	2‴	76.5	3.53
16	74.8	4.45	3‴	77.6	3.58, t (7.2)
17	50.2		4‴	70.7	3.52
18	42.3	2.95, dd (4.5; 14.3)	5‴	66.3	3.33, dd (3.7; 7.9) 3.94, dd (4.5; 11.6)
19	47.9	1.03 2.26, t (13.6)	Rha		
20	31.3		1⁗	101.1	5.16, d (1.8)
21	36.4	1.15, d (11.7) 1.91, d (10.3)	2⁗	71.7	4.04, dd (1.8; 3.3)
22	31.7	1.78	3⁗	81.4	3.87, dd (3.3; 9.4)
23	28.5	1.04, s	4⁗	78.4	3.69, t (9.5)
24	16.9	0.84, s	5⁗	69.1	3.76, dd (6.2; 0.5)
25	16.1	0.95, s	6⁗	18.4	1.26, d (6.2)
26	17.9	0.77, s	Xyl II		
27	27.2	1.36, s	1′′′′′	105.1	4.62, d (7.8)
28	177.2		2′′′′′	75.6	3.16
29	33.3	0.86, s	3′′′′′	78.1	3.85
30	25.0	0.94, s	4′′′′′	71.4	3.47
			5′′′′′	67.0	3.16 3.84, dd (5.5; 11.4)
			Api		
			1′′′′′′	111.9	5.26, d (3.8)
			2′′′′′′	78.2	4.02, d (3.8)
			3′′′′′′	80.0	
			4′′′′′′	74.9	3.74, d (9.5) 4.08, d (9.6)
			5′′′′′′	64.9	3.56, d (1.4)

**Table 2:** ^13^C NMR data for the aglycone moieties of **2–5** (125 MHz, CD_3_OD).

Position	2	3	4	5
1	39.9	39.9	39.9	39.9
2	27.0	27.1	26.9	26.9
3	91.1	91.1	90.9	90.9
4	40.2	40.2	40.8	40.8
5	57.0	57.1	57.1	57.1
6	19.3	19.3	18.5	19.4
7	34.1	34.2	34.3	34.3
8	40.8	40.8	40.8	40.8
9	48.0	48.1	48.1	48.1
10	37.9	37.9	37.8	37.8
11	24.4	24.4	24.5	24.5
12	123.5	123.5	123.6	123.6
13	144.6	144.6	144.7	144.6
14	42.7	42.8	42.7	42.7
15	36.2	36.3	36.5	36.5
16	74.8	74.9	74.6	74.6
17	50.2	50.3	50.2	50.2
18	42.2	42.3	42.2	42.2
19	47.9	47.9	47.9	47.9
20	31.3	31.3	31.3	31.9
21	36.4	36.5	36.4	36.5
22	33.3	33.3	34.3	34.3
23	28.5	28.5	28.6	28.5
24	16.8	16.9	17.0	17.0
25	16.1	16.2	16.2	16.2
26	17.7	17.8	17.8	17.8
27	27.1	27.1	27.1	27.1
28	177.0	177.1	177.0	177.0
29	33.3	33.3	33.3	33.3
30	24.9	25.0	24.9	25.0

**Table 3:** ^1^H and ^13^C NMR data for the sugar moieties of **2–5** (500 and 125 MHz, CD3OD).

Position	2		3		4		5	
	δ_C_	δ_H_	δ_C_	δ_H_	δ_C_	δ_H_	δ_C_	δ_H_
3-*O*-GlcA								
1′	107.0	4.38, d (7.8)	107.0	4.38, d (7.8)	106.9	4.38, d (7.7)	106.9	4.38, d (7.7)
2′	75.9	3.23	75.9	3.23	76.0	3.23	76.0	3.23
3′	77.5	3.35	77.0	3.34	77.9	3.34	77.9	3.34
4′	72.8	3.58	73.0	3.60	72.3	3.90	72.3	3.90
5′	76.6	3.78, d (9.9)	76.2	3.78, d (9.9)	76.1	3.79	76.1	3.79
6′	170.9		170.9		nd		nd	
Ester								
1″	66.1	4.18, td (2.2; 6.4)	66.1	4.18, td (2.2; 6.4)				
2″	31.7	1.65	31.7	1.65				
3″	20.0	1.42	20.0	1.42				
4″	14.0	0.92, t (3.0)	14.0	0.92, t (3.0)				
28-*O*-XylI								
1‴	95.5	5.47, d (6.0)	95.4	5.41, d (6.0)	95.4	5.47, d (6.0)	95.2	5.41, d (6.0)
2‴	76.0	3.56, dd (5.9; 9.1)	76.6	3.53	76.6	3.56	75.5	3.53
3‴	77.8	3.89	77.5	3.90	77.5	3.40	76.0	3.24
4‴	70.9	3.44	70.8	3.47	70.8	3.46	71.1	3.48
5‴	66.7	3.92, dd (4.7; 11.7) 3.33	66.5	3.89	66.5	3.91	66.6	3.92
Rha								
1⁗	98.4	5.22, d (1.8)	101.0	5.23, d (1.8)	98.4	5.24, d (2.0)	101.1	5.23, d (1.8)
2⁗	73.6	5.12, dd (1.8; 3.5)	69.7	4.08, dd (1.8; 3.2)	70.1	4.09, dd (1.8; 3.2)	73.7	5.12, dd (1.7; 3.4)
3⁗	70.3	4.06, dd (3.5; 9.5)	75.8	5.04, dd (3.5; 9.5)	75.9	5.05, dd (3.2; 9.1)	70.8	4.06, dd (3.4; 9.3)
4⁗	83.2	3.52	77.3	3.84	77.9	3.83	83.8	4.53
5⁗	69.0	3.76	69.2	3.82	69.2	3.81	68.9	3.74
6⁗	18.2	1.29, d (6.2)	18.3	1.30, d (6.2)	18.2	1.31, d (6.2)	18.2	1.29, d (6.2)
Xyl II								
1′′′′′	106.5	4.52, d (7.6)	105.6	4.41, d (7.6)	105.5	4.42, d (7.6)	106.7	4.53, d (7.6)
2′′′′′	75.3	3.22	75.8	3.15	75.8	3.15	76.0	3.23
3′′′′′	78.0	3.32	77.8	3.31	78.1	3.39	78.1	3.37
4′′′′′	71.1	3.50	71.3	3.51	71.1	3.46	71.8	3.45
5′′′′′	67.3	3.85 3.17	67.0	3.83 3.19	67.2	3.87 3.32	67.2	3.87 3.32
OAc	172.2		172.5		172.1		172.0	
	20.9	2.09, s	21.3	2.10, s	21.3	2.11, s	21.3	2.10, s

**Table 4:** ^13^C NMR data for the aglycone moieties of **6–8** (200 MHz, CD_3_OD).

Position	6	7	8
1	39.9	39.9	39.9
2	26.7	26.8	26.9
3	90.8	90.8	90.7
4	40.2	40.2	40.2
5	57.1	57.1	57.2
6	19.3	19.3	19.4
7	34.2	34.3	34.5
8	40.8	40.9	40.8
9	48.1	48.2	48.2
10	37.9	37.9	37.9
11	24.5	24.5	24.5
12	123.6	123.6	123.7
13	145.5	145.7	145.6
14	42.8	42.8	42.8
15	36.4	36.5	36.6
16	74.9	74.6	74.7
17	50.2	50.3	50.4
18	42.2	42.2	42.3
19	47.9	47.8	47.8
20	31.3	31.3	31.3
21	36.4	36.4	36.4
22	31.8	31.8	32.0
23	28.6	28.5	28.5
24	17.1	17.0	17.0
25	16.2	16.2	16.2
26	17.9	17.8	17.8
27	27.2	27.2	27.2
28	177.0	177.1	177.2
29	33.4	33.4	33.4
30	24.9	25.0	25.1

**Table 5:** ^1^H and ^13^C NMR data for the sugar moieties of **6–8** (800 and 200 MHz, CD_3_OD).

Position	6		7		8	
	δ_C_	δ_H_	δ_C_	δ_H_	δ_C_	δ_H_
3-*O*-Glc A						
1′	106.7	4.34, d (7.8)	106.7	4.34, d (7.8)	106.7	4.34, d (7.8)
2′	75.2	3.22–3.24	75.5	3.22–3.24	75.4	3.22–3.24
3′	78.0	3.37	78.0	3.37	78.0	3.37
4′	73.7	3.47, t (9.3)	73.7	3.47, t (9.3)	73.8	3.47, t (9.3)
5′	76.8	3.54–3.58	76.8	3.54–3.58	76.8	3.54–3.58
6′	nd		nd		nd	
28-*O*-Xyl						
1″	95.5	5.52, d (6.0)	95.3	5.48, d (6.0)	95.4	5.44, d (6.0)
2″	76.9	3.56	76.9	3.56	76.8	3.54–3.58
3″	75.8	3.56–3.58	75.8	3.56–3.58	75.8	3.56–3.58
4″	70.8	3.51–3.53	70.8	3.51–3.53	70.8	3.51–3.53
5″	66.5	3.31–3.33 3.92–3.94	66.5	3.31–3.33 3.92–3.94	66.6	3.31–3.33 3.92–3.94
Rha						
1‴	101.5	5.33, d (2.0)	101.5	5.23, d (1.9)	101.6	5.15, d (1.8)
2‴	73.9	5.12, d (2.1)	70.2	4.07, dd (1.9; 3.3)	70.2	3.89
3‴	70.8	3.51–3.53	75.8	4.87	72.2	3.95
4‴	70.9	3.59	70.9	3.59	75.4	4.93
5‴	70.5	3.65–3.68	70.5	3.65–3.68	68.2	3.35
6‴	18.0	1.26, d (6.5)	18.3	1.29, d (6.7)	18.2	1.27, d (6.7)
OAc	172.4		172.7		172.6	
	20.5	2.08, s	21.1	2.10, s	21.2	2.09, s

## Data Availability

The data presented in this study are available in the main article and the supplementary materials.

## Supplementary Materials

The following supporting information can be downloaded at: https://www.mdpi.com/article/10.3390/metabo12111018/s1, Figure S1: HRFABMS spectrum of compound **1**, Figure S2: IR spectrum of compound **1**, Figure S3: ^1^H NMR (600 MHz, CD3OD) spectrum of compound **1**, Figure S4: ^13^C NMR (150 MHz, CD3OD) spectrum of compound **1**, Figure S5: DEPT 135 spectrum of compound **1**, Figure S6: DEPT 90 spectrum of compound **1**, Figure S7: ^1^H−^1^H COSY spectrum of compound **1**, Figure S8: ROESY spectrum of compound **1**, Figure S9: TOCSY spectrum of compound **1**. Figure S10: HSQC spectrum of compound **1**, Figure S11: HMBC spectrum of compound **1**, Figure S12: HRFABMS spectrum of compound **2/3**, Figure S13: IR spectrum of compound **2/3**, Figure S14: ^1^H NMR (600 MHz, CD3OD) spectrum of compound **2/3**, Figure S15: ^13^C NMR (150 MHz, CD3OD) spectrum of compound **2/3**, Figure S16: DEPT 135 spectrum of compound **2/3**, Figure S17: DEPT 90 spectrum of compound **2/3**, Figure S18: ^1^H−^1^H COSY spectrum of compound **2/3**, Figure S19: NOESY spectrum of compound **2/3**, Figure S20: TOCSY spectrum of compound **2/3**, Figure S21: HSQC spectrum of compound **2/3**, Figure S22: HMBC spectrum of compound **2/3**, Figure S23: HRFABMS spectrum of compound **4/5**, Figure S24: ESI-MS2 spectrum of *m/z* = 1099.6 of compound **4/5**, Figure S25: ESI-MS3 spectrum of *m/z* = 647.4 of compound **4/5**, Figure S26: IR spectrum of compound **4/5**, Figure S27: ^1^H NMR (600 MHz, CD3OD) spectrum of compound **4/5**, Figure S28: ^13^C NMR (150 MHz, CD3OD) spectrum of compound **4/5**, Figure S29: DEPT 135 spectrum of compound **4/5**, Figure S30: DEPT 90 spectrum of compound **4/5**, Figure S31: ^1^H−^1^H COSY spectrum of compound **4/5**, Figure S32: NOESY spectrum of compound **4/5**, Figure S33: TOCSY spectrum of compound **4/5**, Figure S34: HSQC spectrum of compound **4/5**, Figure S35: HMBC spectrum of compound **4/5**, Figure S36: HRFABMS spectrum of compounds **6–8**, Figure S37: IR spectrum of compound **6–8**, Figure S38: ^1^H NMR (800 MHz, CD3OD) spectrum of compound **6–8**, Figure S39: ^13^C NMR (200 MHz, CD3OD) spectrum of compound **6–8**, Figure S40: DEPT 135 spectrum of compound **6–8**, Figure S41: DEPT 90 spectrum of compound **6–8**, Figure S42: ^1^H−^1^H COSY spectrum of compound **6–8**, Figure S43: NOESY spectrum of compound **6–8**, Figure S44: HSQC spectrum of compound **6–8**, Figure S45: HBMC spectrum of compound **6–8**, Figure S46: HRFABMS spectrum of compound **9**, Figure S47: ^1^H NMR (600 MHz, CD3OD) spectrum of compound **9**, Figure S48: ^13^C NMR (150 MHz, CD3OD) spectrum of compound **9**, Figure S49: DEPT 135 spectrum of compound **9**, Figure S50: DEPT 90 spectrum of compound **9**, Figure S51: Cell viability of all tested compounds.
