# Supplementary material for: Echinocystic Acid Bidesmoside Saponins from Microglossa afzelii O. Hoffm and Their Cytotoxic Activity against the CAL-27 Oral Squamous Carcinoma Cell Line
Source: Metabolites. 2022 Oct 25;12(11):1018. doi: 10.3390/metabo12111018 (PMC9694296; doi:10.3390/metabo12111018)
Supplement: Supplementary file 1 [file metabolites-12-01018-s001.zip › metabolites-1955665-supplementary.pdf]

Supporting information

Echinocystic Acid Bidesmoside Saponins from *Microglossa afzelii* O. Hoffm and Their Cytotoxic Activity against the CAL-27 Oral Squamous Carcinoma Cell Line

Billy Toussie Tchegnitegni [1,2,*], Tehmina Ahmed [3], Francis Tatong Ngouafong [1], Viviane Flore Kamlo Kamso [4], Rémy Bertrand Teponno [1], Bruno Ndjakou Lenta [5], Léon Azefack Tapondjou [1], Arslan Ali [3] and Syed Ghulam Musharraf [2,*]

[1] Department of Chemistry, Faculty of Science, University of Dschang, Dschang P.O. Box 67, Cameroon
[2] HEJ, Research Institute of Chemistry, International Center for Chemical and Biological Sciences, University of Karachi, Karachi 75270, Pakistan
[3] Panjwani Center for Molecular Medicine and Drug Research, International Center for Chemical and Biological Sciences, University of Karachi, Karachi 75270, Pakistan
[4] Department of Organic Chemistry, Faculty of Science, University of Yaoundé I, Yaoundé P.O. Box 812, Cameroon
[5] Department of Chemistry, Higher Teacher Training College, University of Yaoundé I, Yaoundé P.O. Box 47, Cameroon
* Correspondence: billytoussie@yahoo.fr (B.T.T.); musharraf1977@yahoo.com (S.G.M.)

TABLE OF CONTENTS

Figure S1. HRFABMS spectrum of compound **1**

Figure S2. IR spectrum of compound **1**

Figure S3. ^{1}H NMR (600 MHz, CD_3OD) spectrum of compound **1**

Figure S4. ^{13}C NMR (150 MHz, CD_3OD) spectrum of compound **1**

Figure S5. DEPT 135 spectrum of compound **1**

Figure S6. DEPT 90 spectrum of compound **1**

Figure S7. ^{1}H–^{1}H COSY spectrum of compound **1**

Figure S8. ROESY spectrum of compound **1**

Figure S9. TOCSY spectrum of compound **1**

Figure S10. HSQC spectrum of compound **1**

Figure S11. HMBC spectrum of compound **1**

Figure S12. HRFABMS spectrum of compound **2/3**

Figure S13. IR spectrum of compound **2/3**

Figure S14. ^{1}H NMR (600 MHz, CD_3OD) spectrum of compound **2/3**

Figure S15. ^{13}C NMR (150 MHz, CD_3OD) spectrum of compound **2/3**

Figure S16. DEPT 135 spectrum of compound **2/3**

Figure S17. DEPT 90 spectrum of compound **2/3**

Figure S18. ^{1}H–^{1}H COSY spectrum of compound **2/3**

Figure S19. NOESY spectrum of compound **2/3**

Figure S20. TOCSY spectrum of compound **2/3**

Figure S21. HSQC spectrum of compound **2/3**

Figure S22. HMBC spectrum of compound **2/3**

Figure S23. HRFABMS spectrum of compound **4/5**

Figure S24. ESI-MS^2 spectrum of m/z = 1099.6 of compound **4/5**

Figure S25. ESI-MS^3 spectrum of m/z = 647.4 of compound **4/5**

Figure S26. IR spectrum of compound **4/5**

Figure S27. ^1H NMR (600 MHz, CD_3OD) spectrum of compound **4/5**

Figure S28.^{13}C NMR (150 MHz, CD_3OD) spectrum of compound **4/5**

Figure S29. DEPT 135 spectrum of compound **4/5**

Figure S30. DEPT 90 spectrum of compound **4/5**

Figure S31. ^1H–^1H COSY spectrum of compound **4/5**

Figure S32. NOESY spectrum of compound **4/5**

Figure S33. TOCSY spectrum of compound **4/5**

Figure S34. HSQC spectrum of compound **4/5**

Figure S35. HMBC spectrum of compound **4/5**

Figure S36. HRFABMS spectrum of compounds **6−8**

Figure S37. IR spectrum of compound **6−8**

Figure S38. ^1H NMR (800 MHz, CD_3OD) spectrum of compound **6−8**

Figure S39. ^{13}C NMR (200 MHz, CD_3OD) spectrum of compound **6−8**

Figure S40. DEPT 135 spectrum of compound **6−8**

Figure S41. DEPT 90 spectrum of compound **6−8**

Figure S42. ^{1}H–^{1}H COSY spectrum of compound **6−8**

Figure S43. NOESY spectrum of compound **6−8**

Figure S44. HSQC spectrum of compound **6−8**

Figure S45. HBMC spectrum of compound **6−8**

Figure S46. HRFABMS spectrum of compound **9**

Figure S47. ^{1}H NMR (600 MHz, CD_3OD) spectrum of compound **9**

Figure S48. ^{13}C NMR (150 MHz, CD_3OD) spectrum of compound **9**

Figure S49. DEPT 135 spectrum of compound **9**

Figure S50. DEPT 90 spectrum of compound **9**

Figure S51. Cell viability of all tested compounds.

HEJ-MASS LAB -ICCBS | JEOL HX 110 MASS SPECTROMETER (FAB-HR)

Measured MASS	Theoretical MASS	Delta [ppm]	Delta [mmu]	RDB	Composition
1245.6211	1245.6209	0.1	0.2	22.5	C68 H93 O21
	1245.6268	-4.6	-5.7	13.5	C61 H97 O26
	1245.6057	12.4	15.4	18.5	C64 H93 O24
	1245.6421	-16.8	-21	17.5	C65 H97 O23
	1245.6573	-29.1	-36.2	21.5	C69 H97 O20

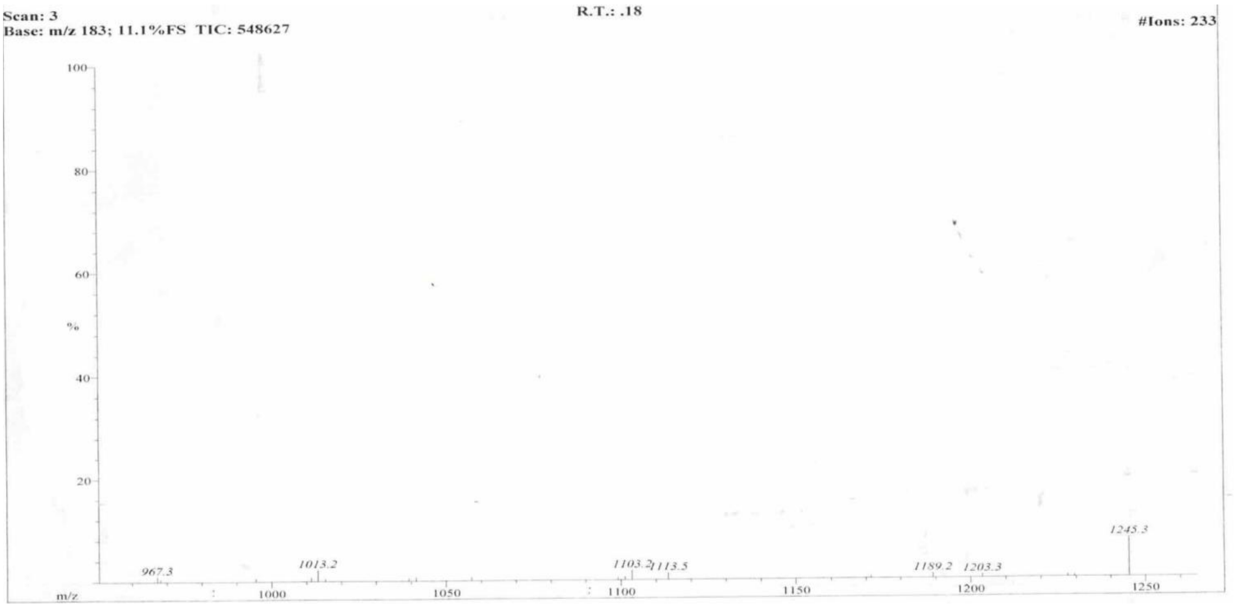

Figure S1. HRFABMS spectrum of compound **1**

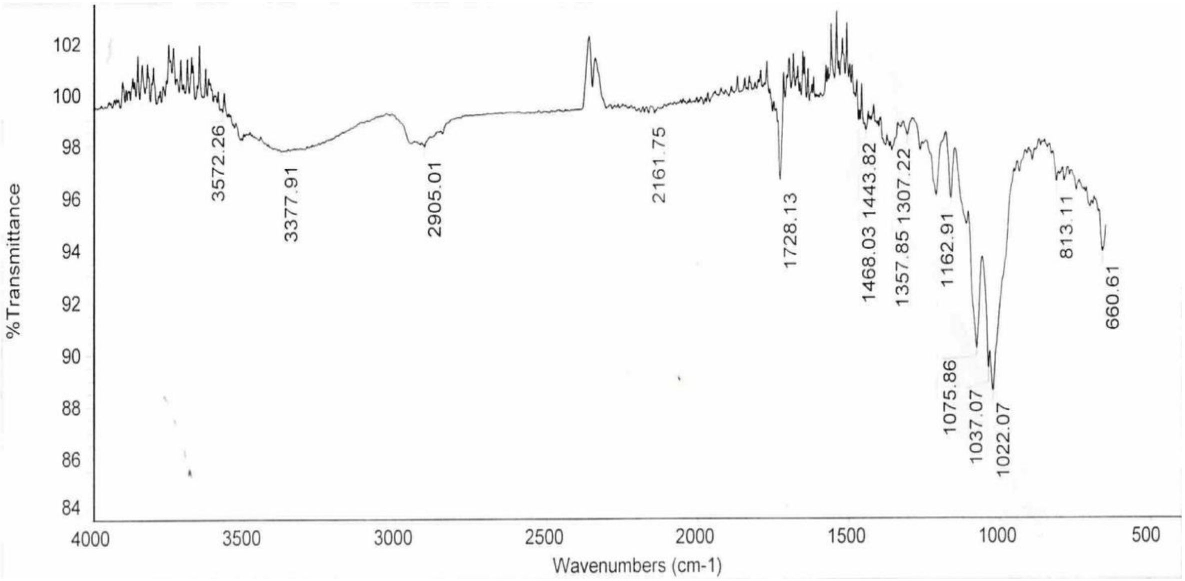

Figure S2. IR spectrum of compound **1**

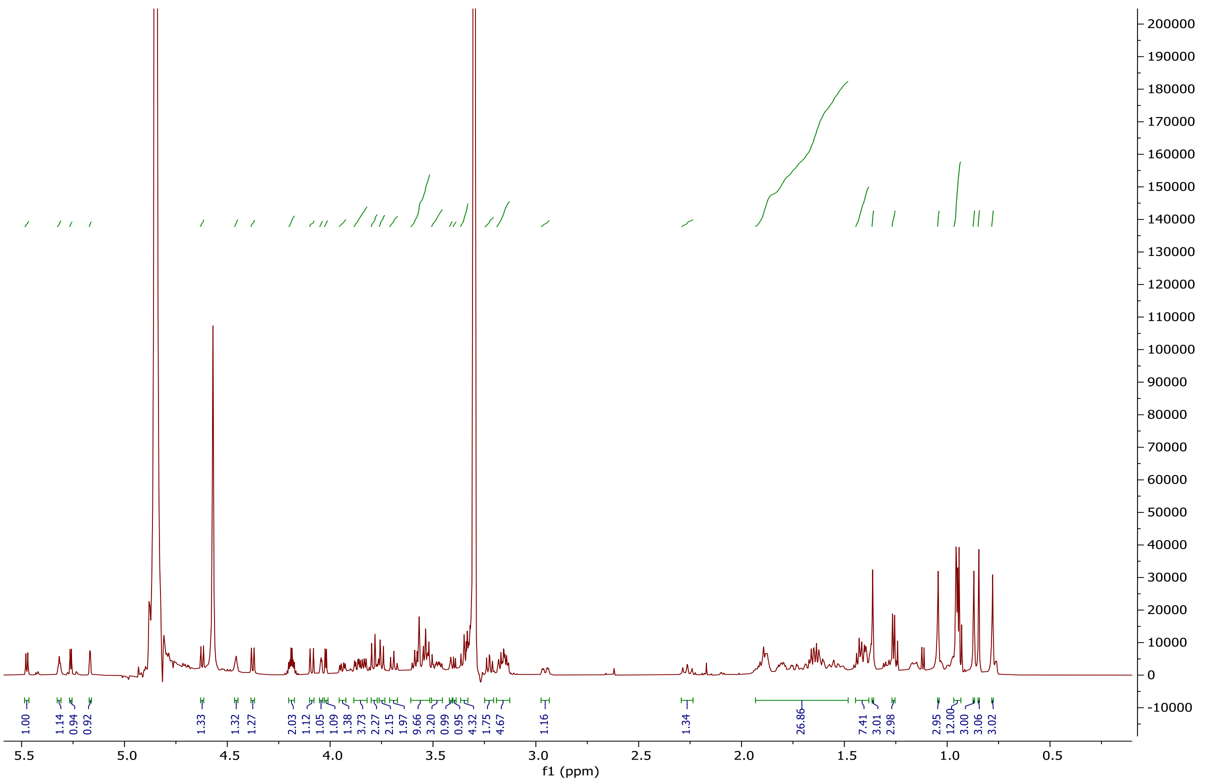

Figure S3. ^{1}H NMR (600 MHz, CD_3OD) spectrum of compound **1**

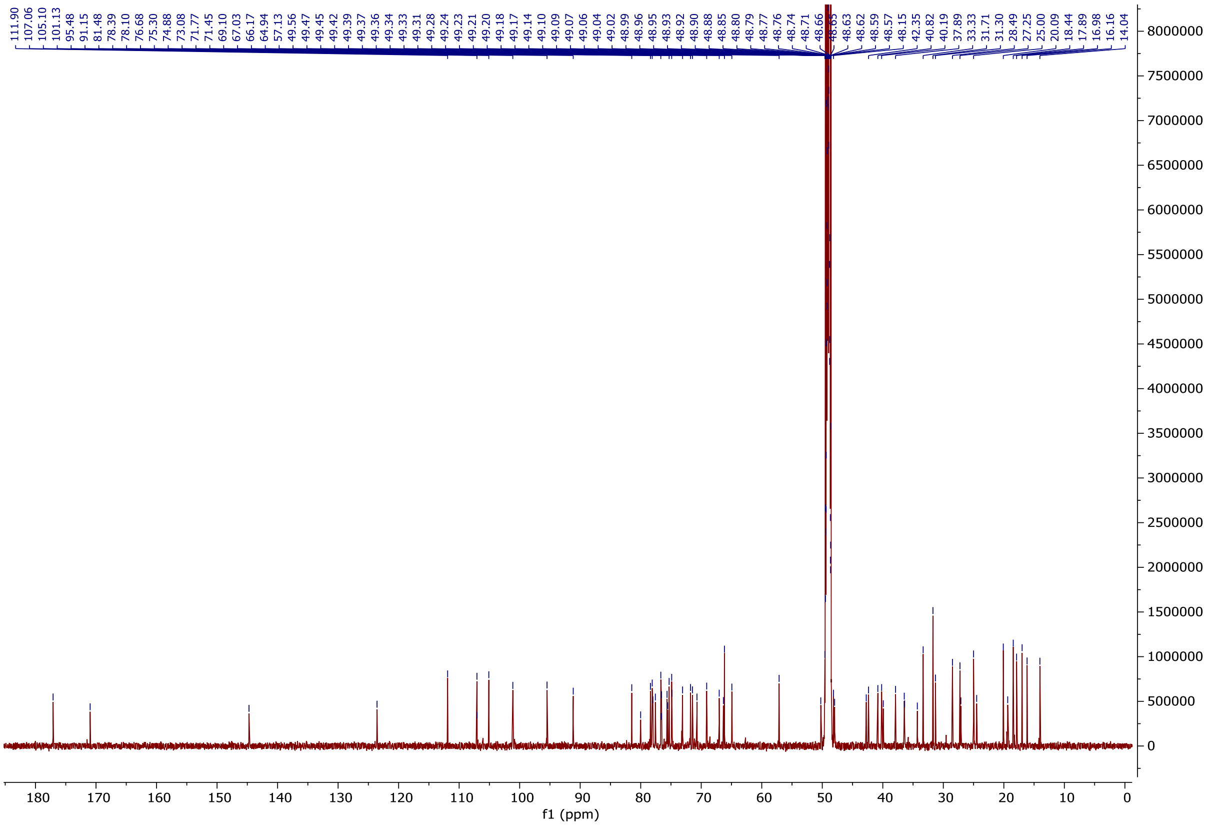

Figure S4. ^{13}C NMR (150 MHz, CD_3OD) spectrum of compound **1**

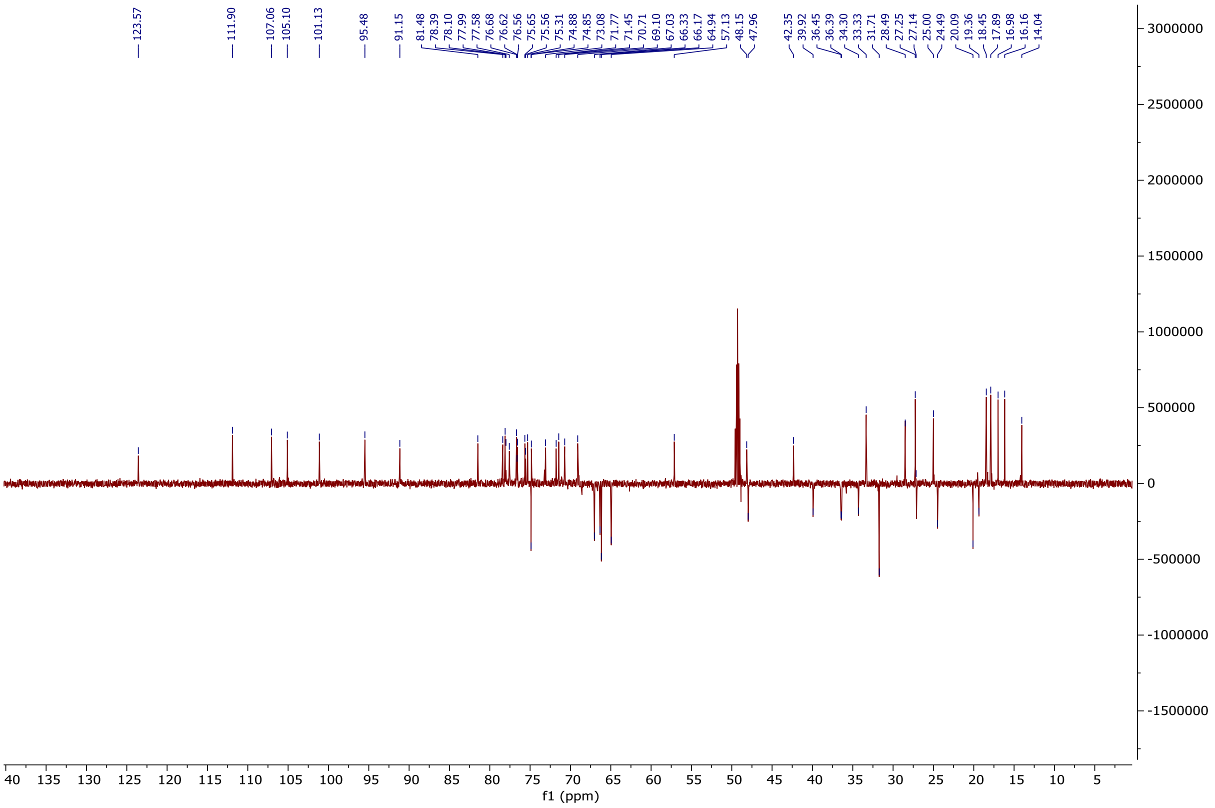

Figure S5. DEPT 135 spectrum of compound **1**

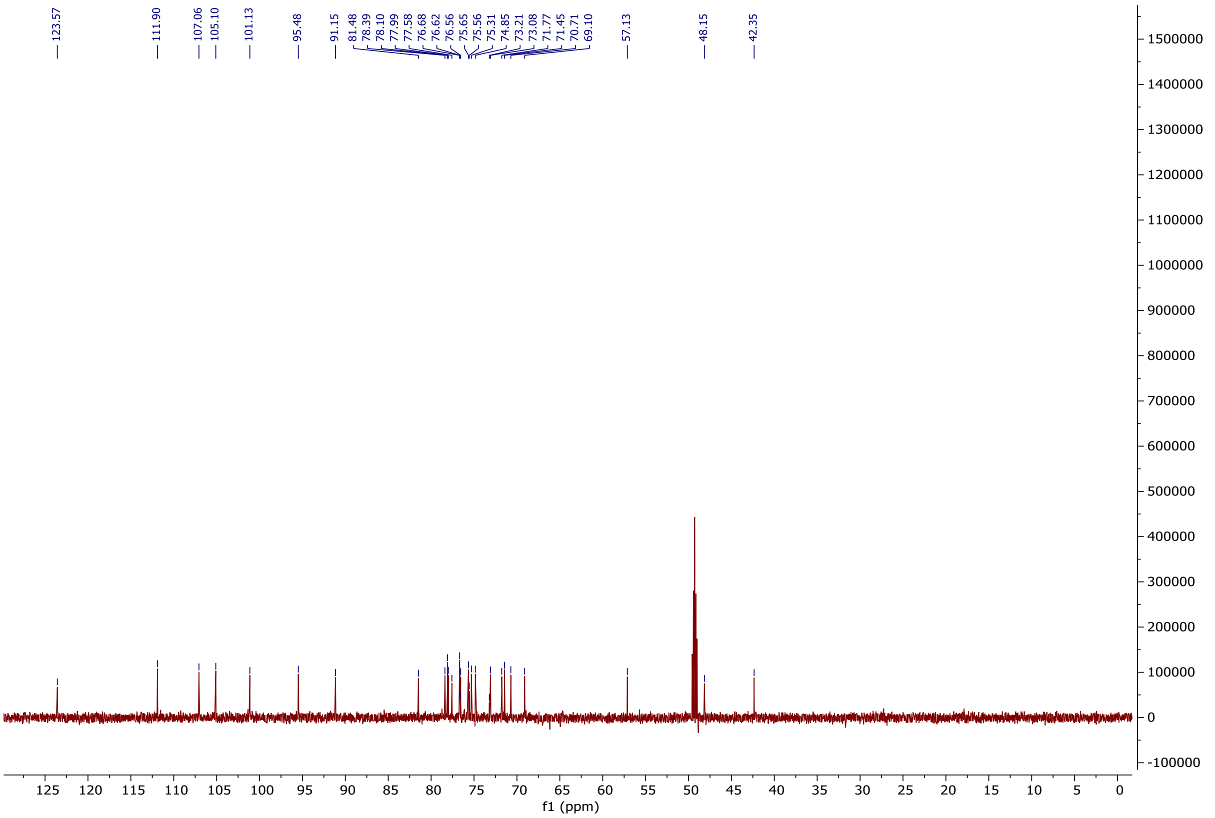

Figure S6: DEPT 90 spectrum of compound **1**

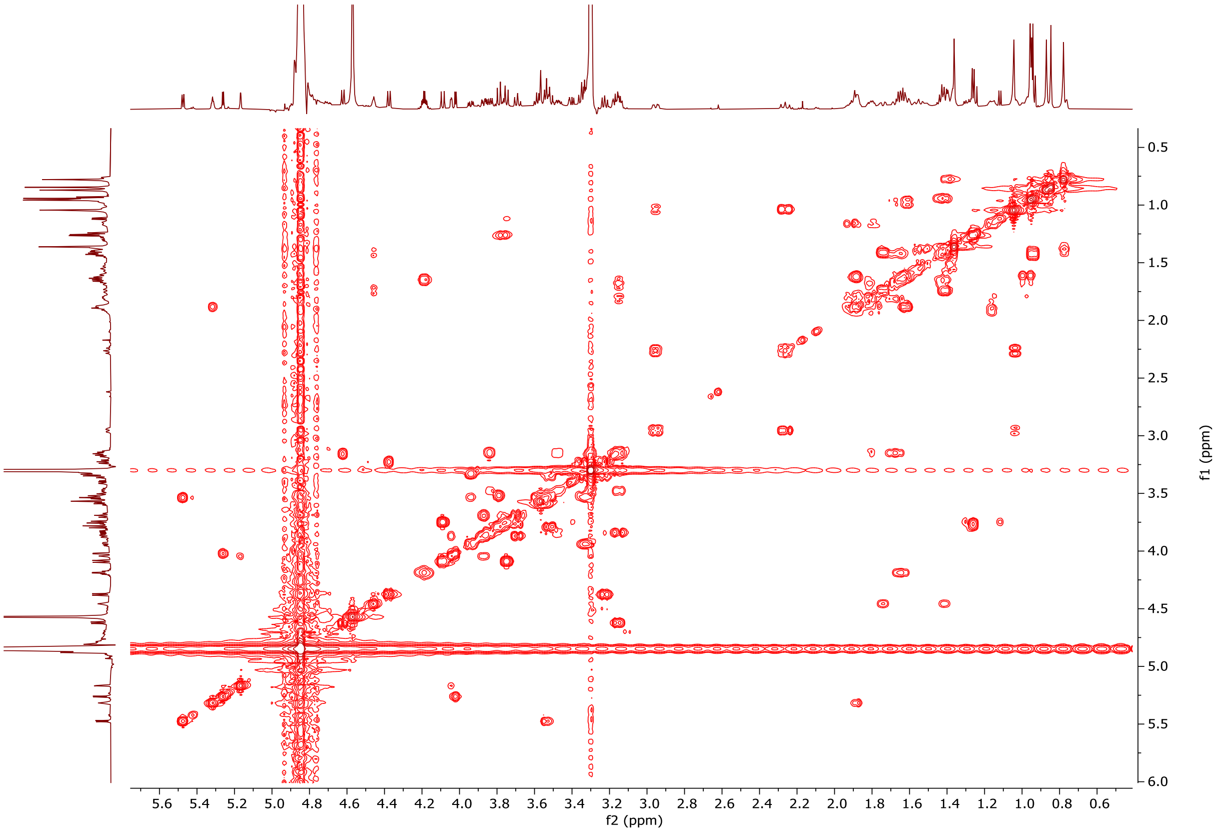

Figure S7. ^{1}H–^{1}H COSY spectrum of compound **1**

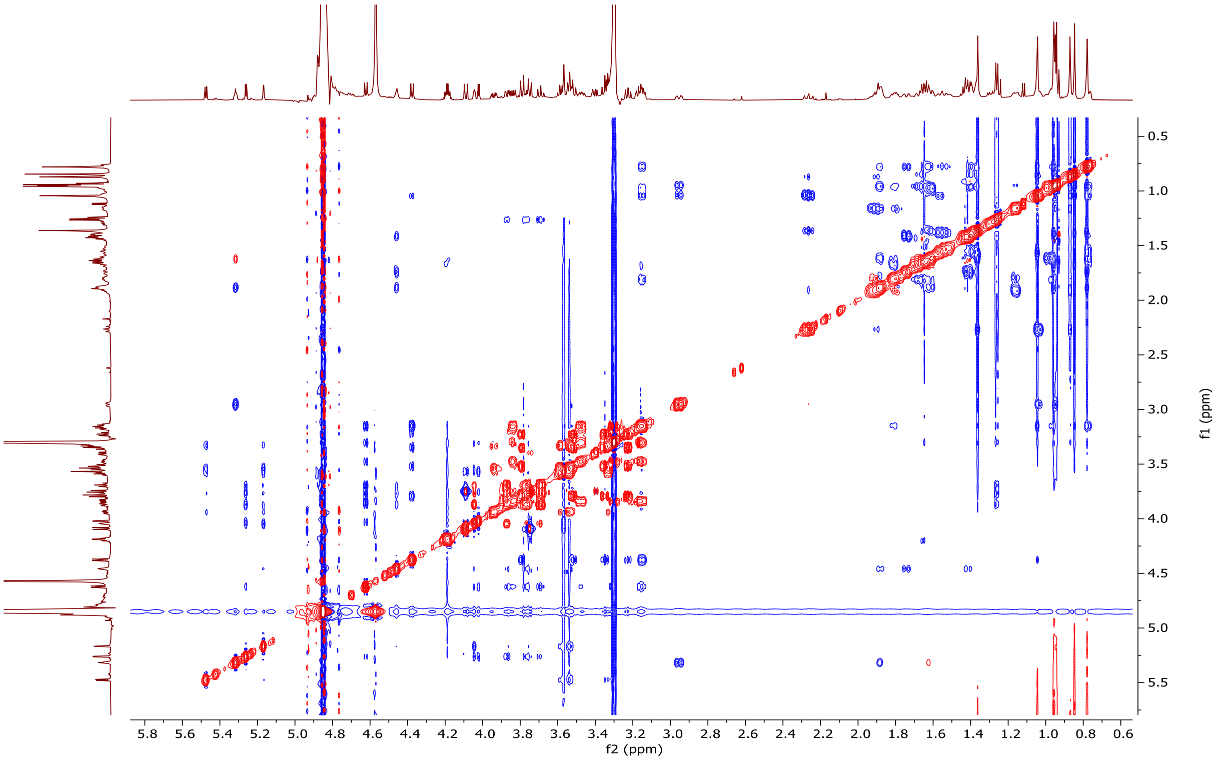

Figure S8. ROESY spectrum of compound **1**

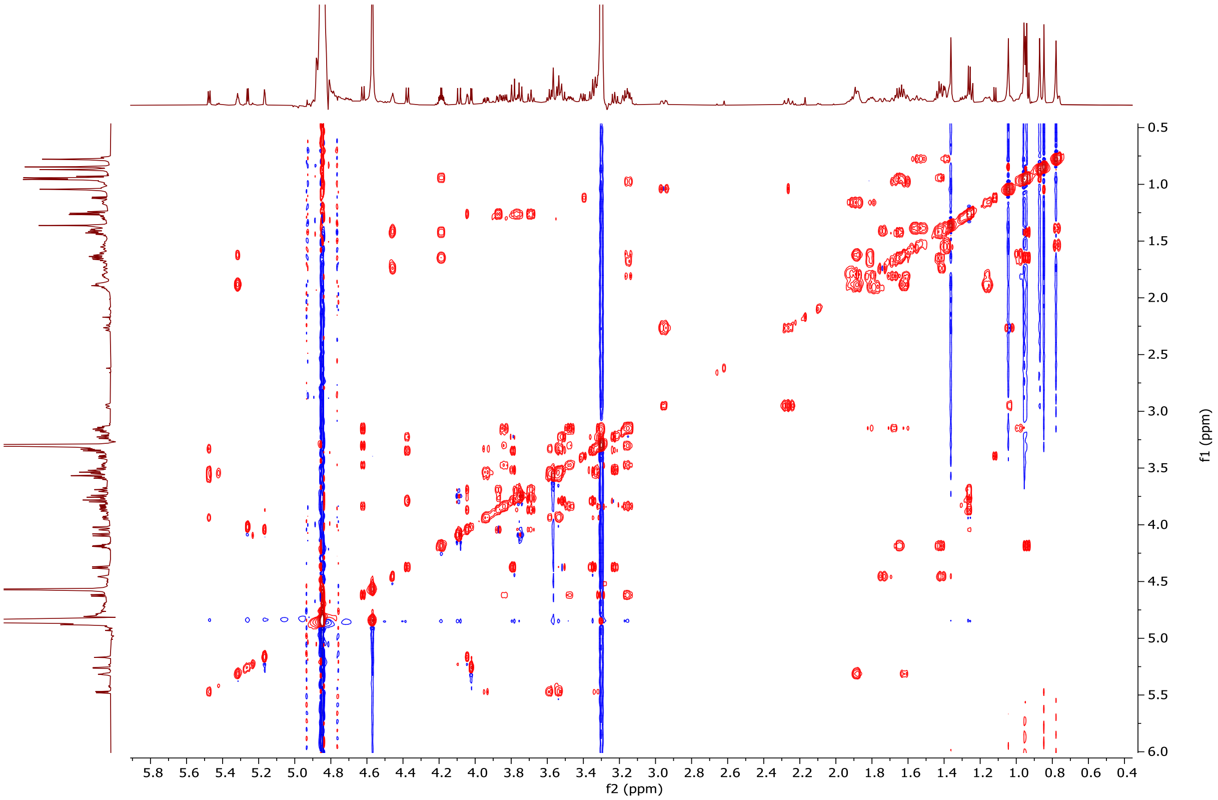

Figure S9. TOCSY spectrum of compound **1**

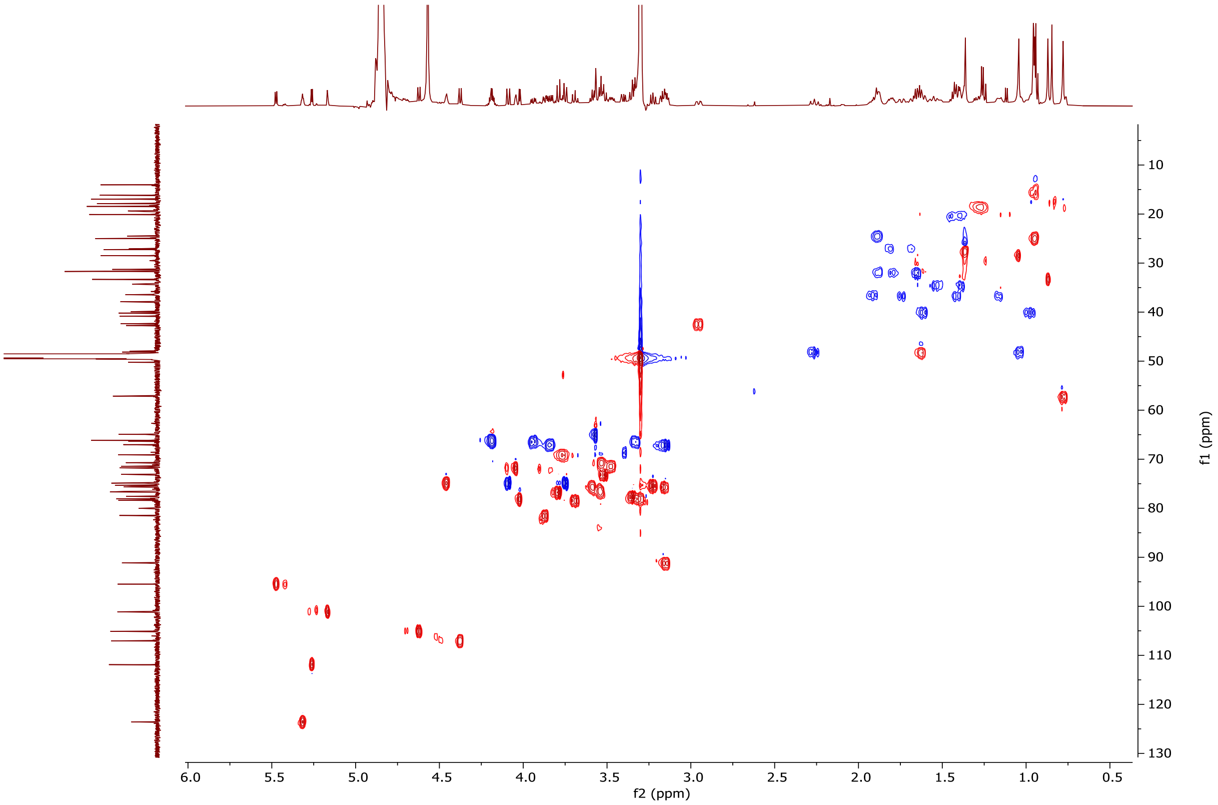

Figure S10. HSQC spectrum of compound **1**

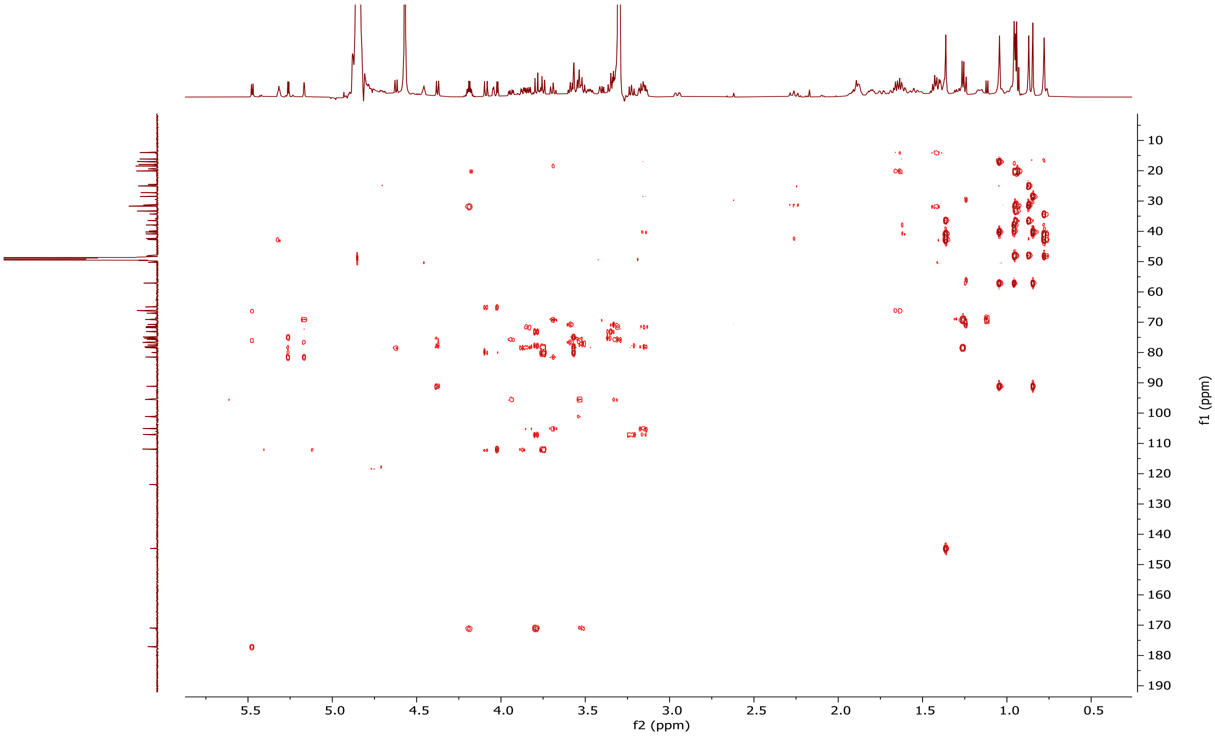

Figure S11. HMBC spectrum of compound **1**

HEJ-MASS LAB -ICCBS

JEOL HX 110 MASS SPECTROMETER (FAB-HR)

Measured MASS	Theoretical MASS	Delta	Delta	RDB	Composition
1155.5981	1155.5951	2.6	3	13.5	C58 H91 O23
	1155.6045	-5.5	-6.4	26.5	C69 H87 O15
	1155.5892	7.7	8.9	22.5	C65 H87 O18
	1155.6104	-10.6	-12.3	17.5	C62 H91 O20

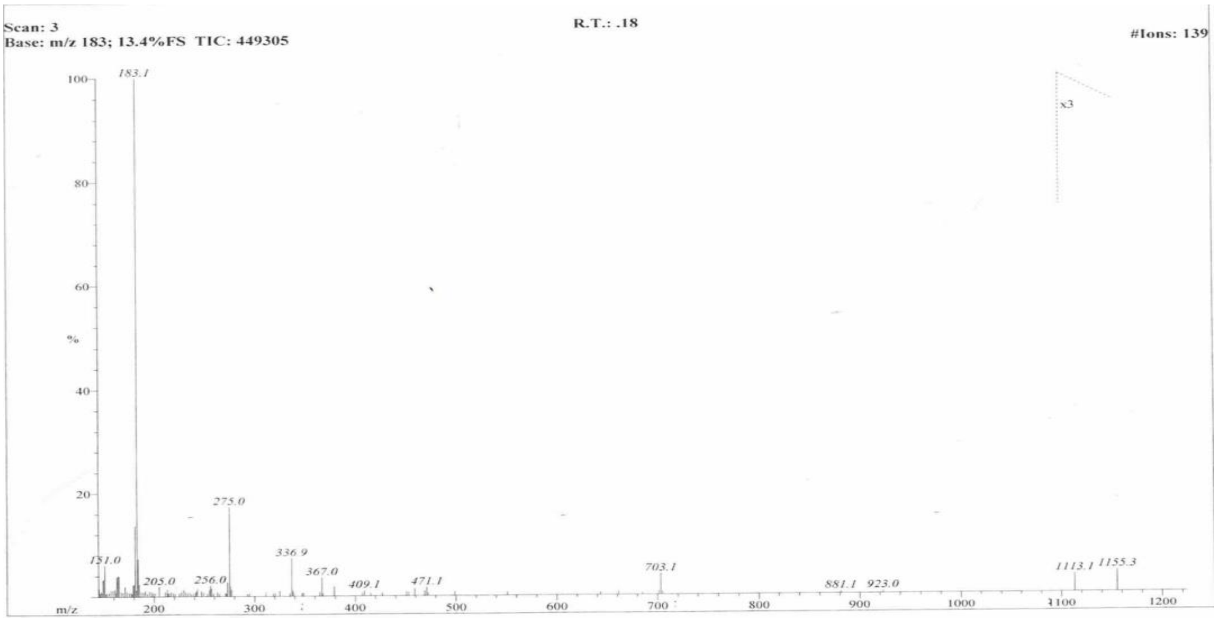

Figure S12. HRFABMS spectrum of compound **2/3**

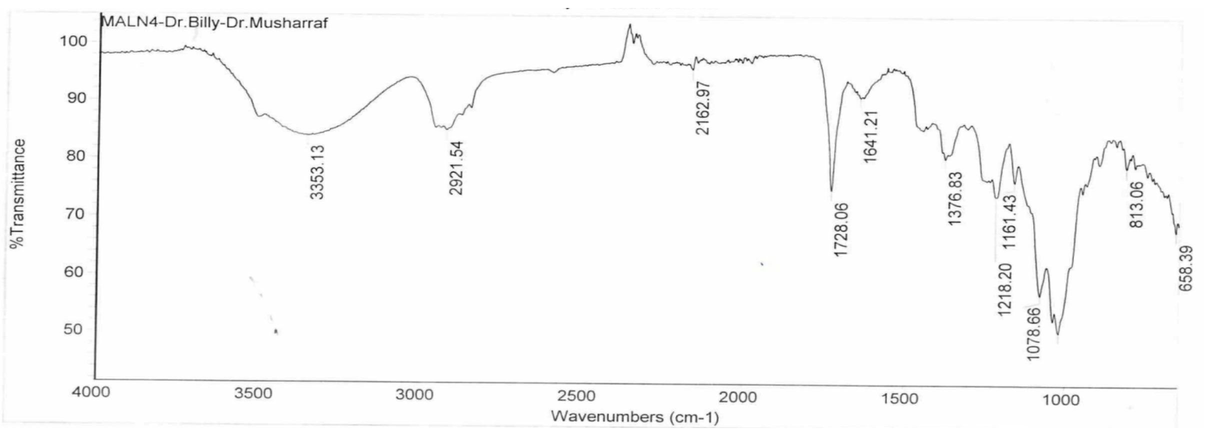

Figure S13. IR spectrum of compound **2/3**

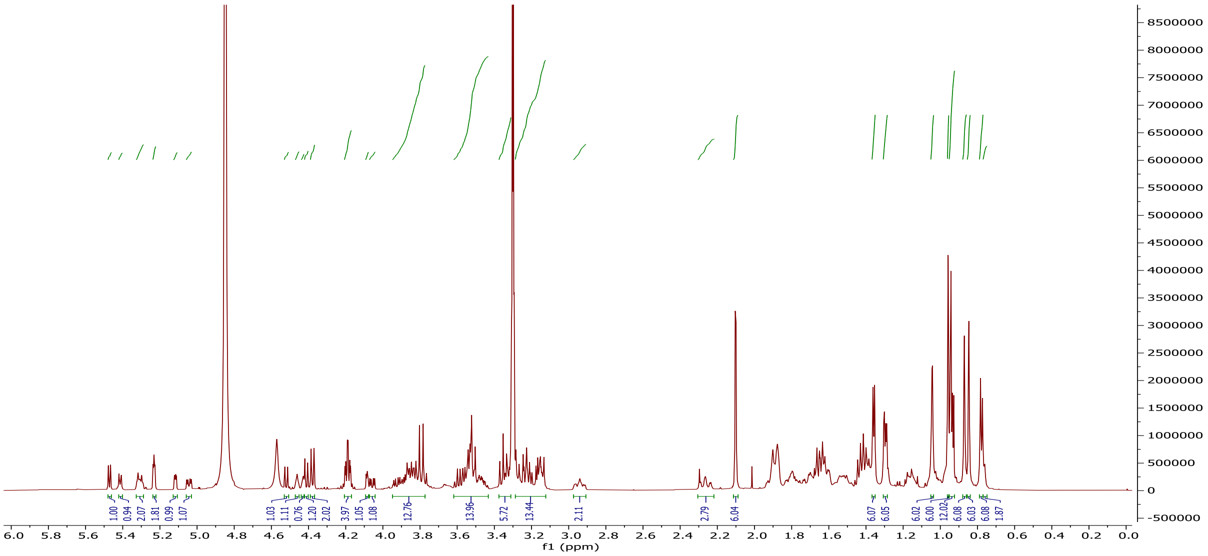

Figure S14. ^{1}H NMR (600 MHz, CD_3OD) spectrum of compound **2/3**

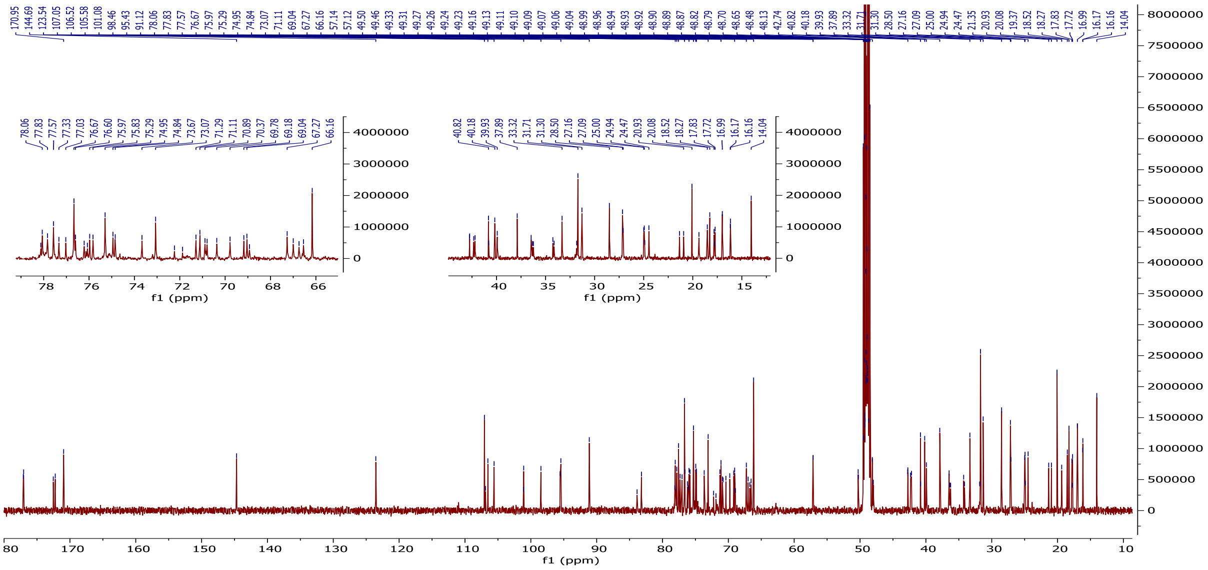

Figure S15. ^{13}C NMR (150 MHz, CD_3OD) spectrum of compound **2/3**

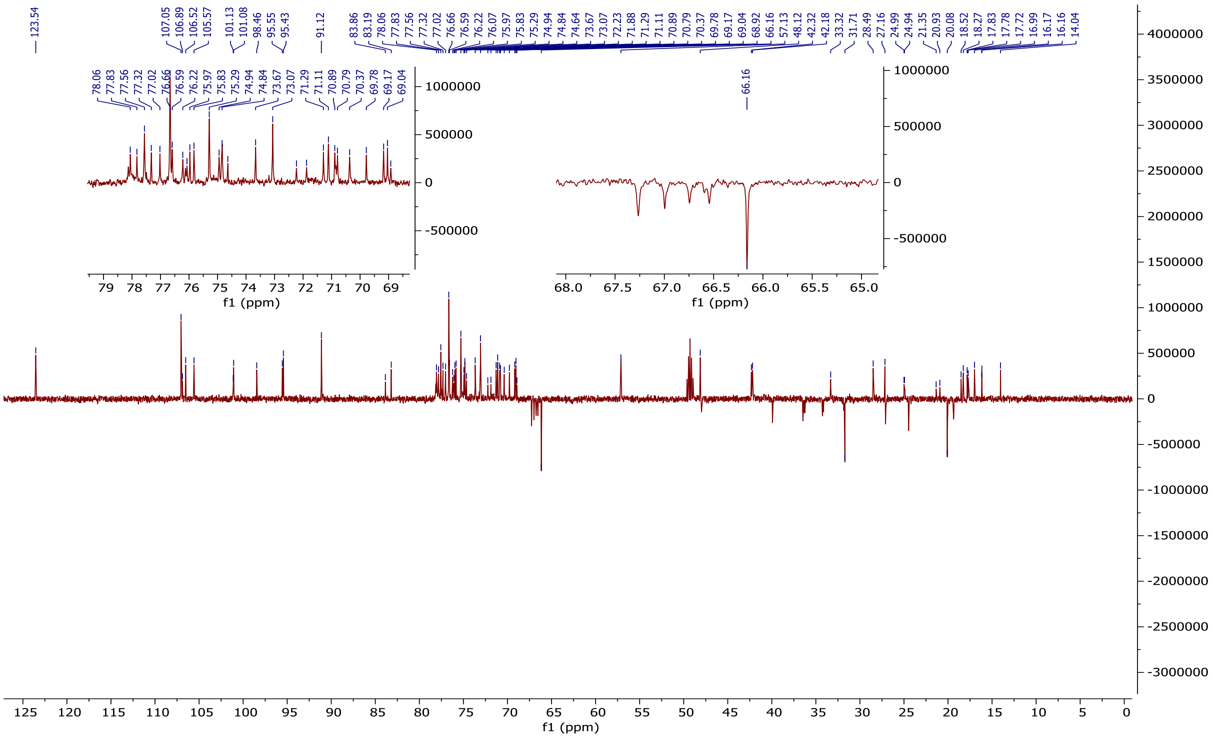

Figure S16. DEPT 135 spectrum of compound **2/3**

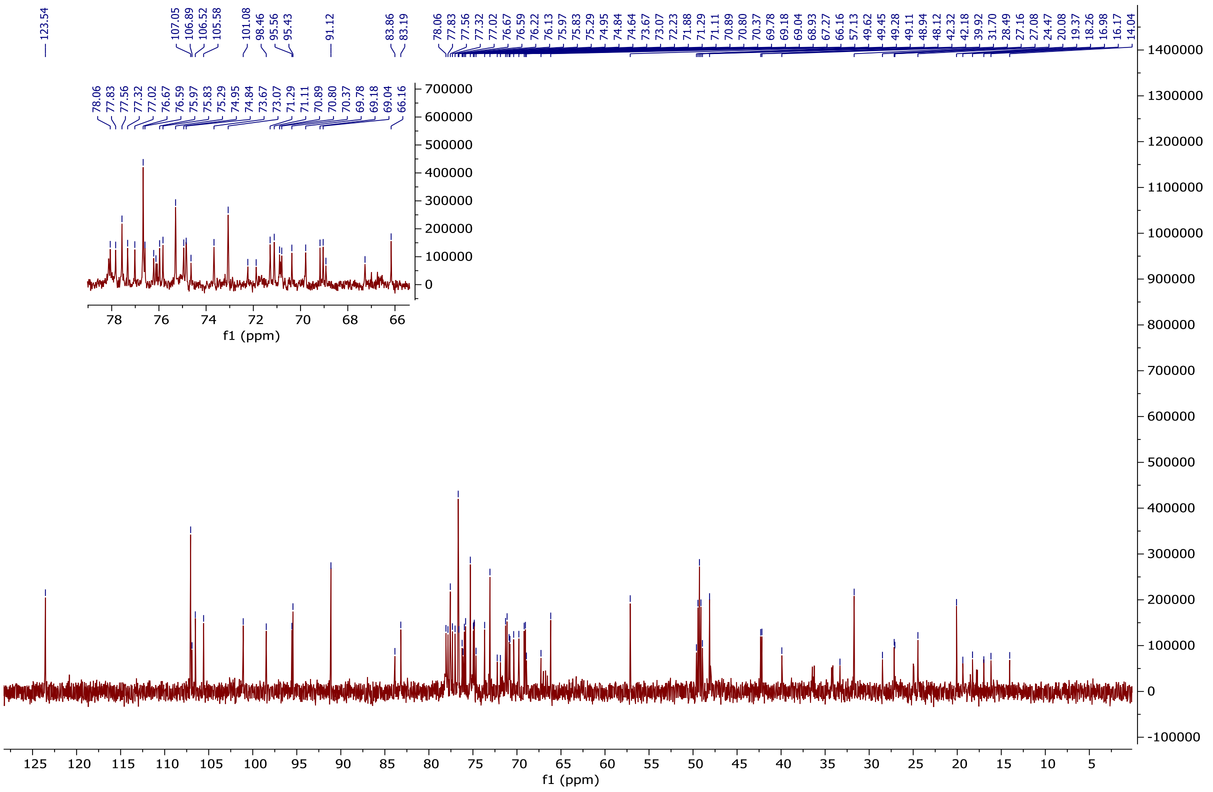

Figure S17. DEPT 90 spectrum of compound **2/3**

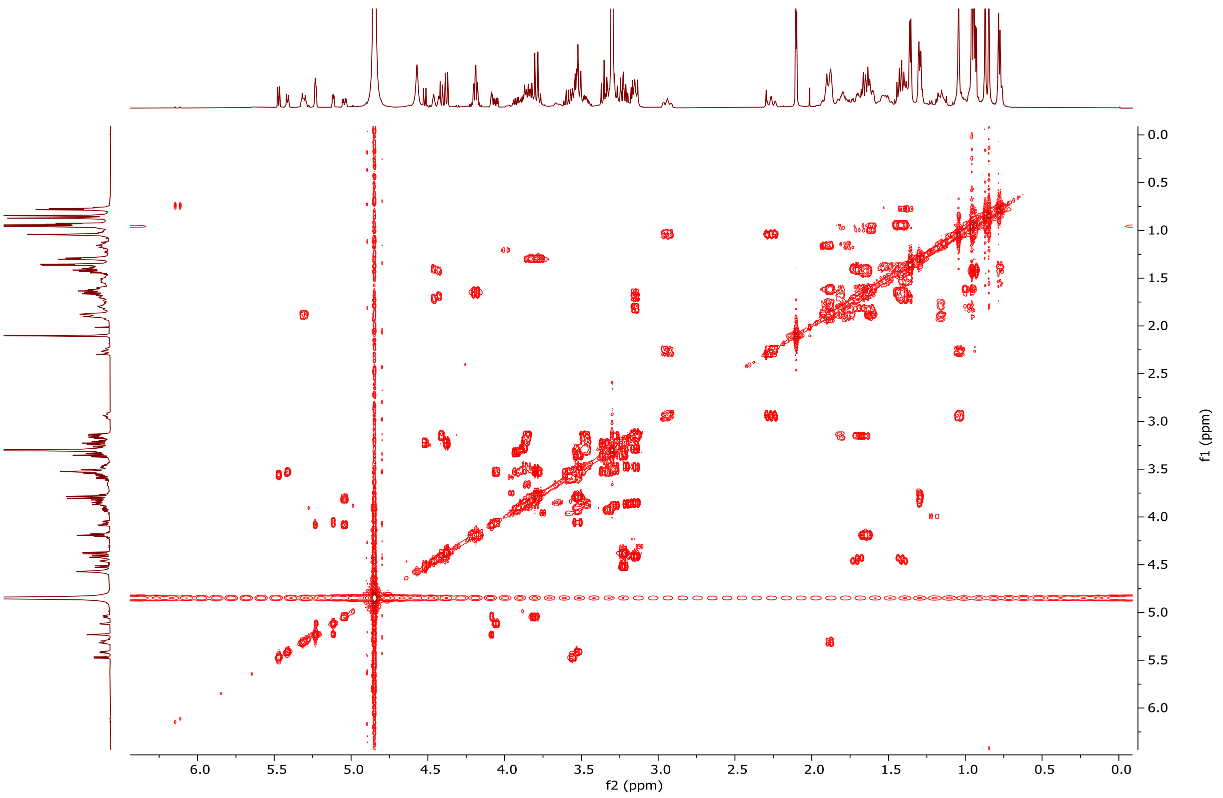

Figure S18. ^{1}H–^{1}H COSY spectrum of compound **2/3**

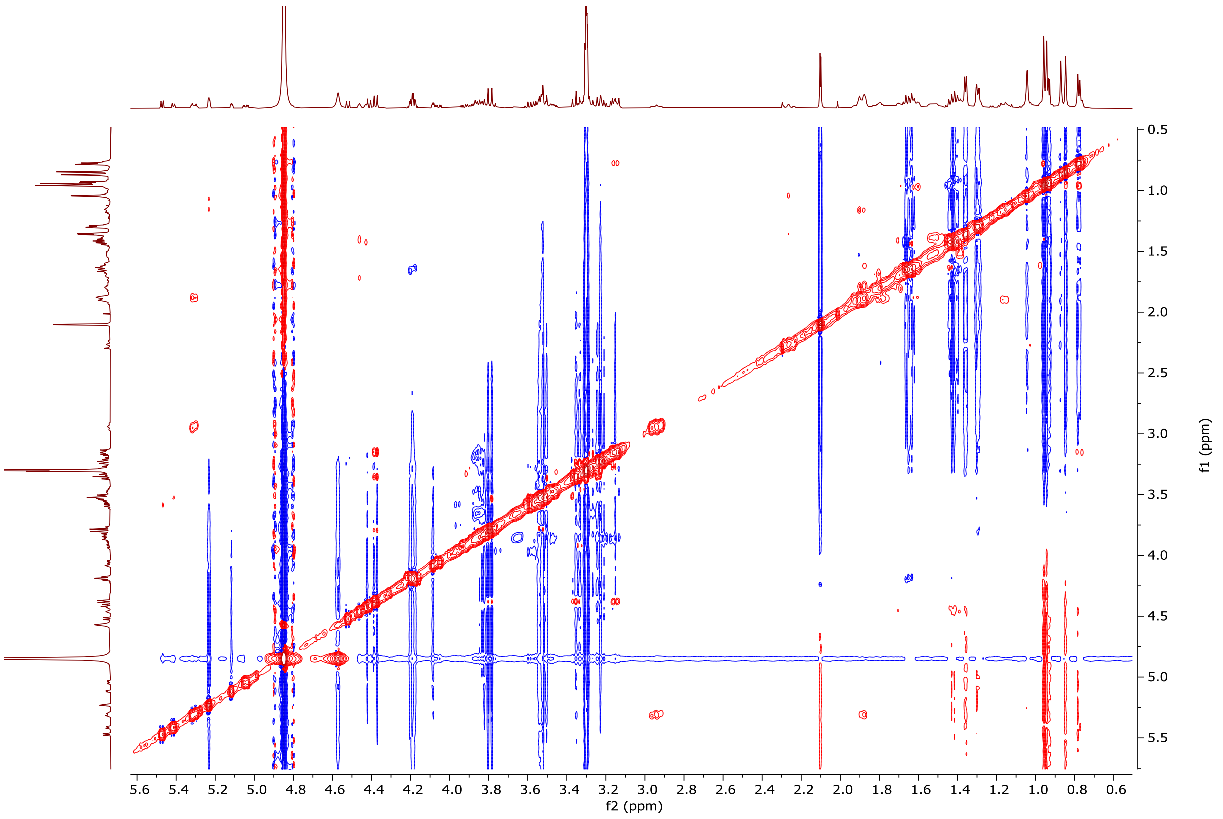

Figure S19. NOESY spectrum of compound **2/3**

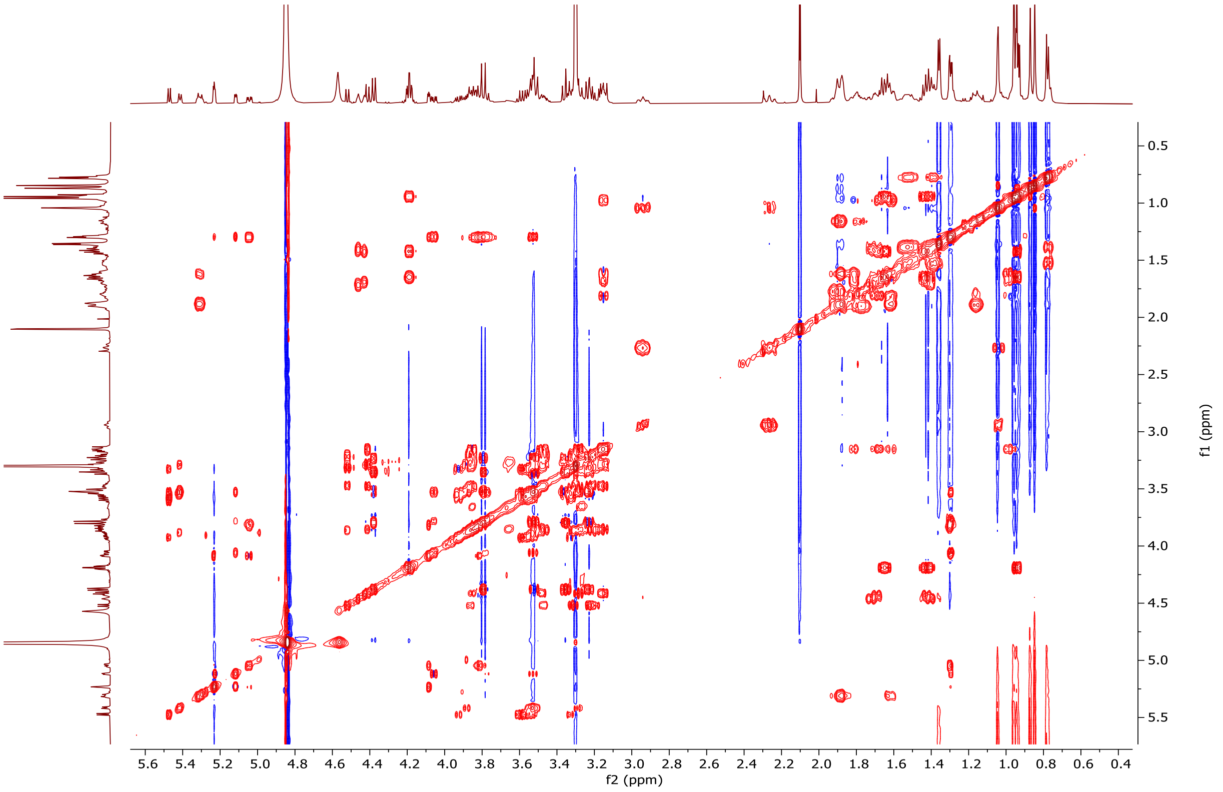

Figure S20. TOCSY spectrum of compound **2/3**

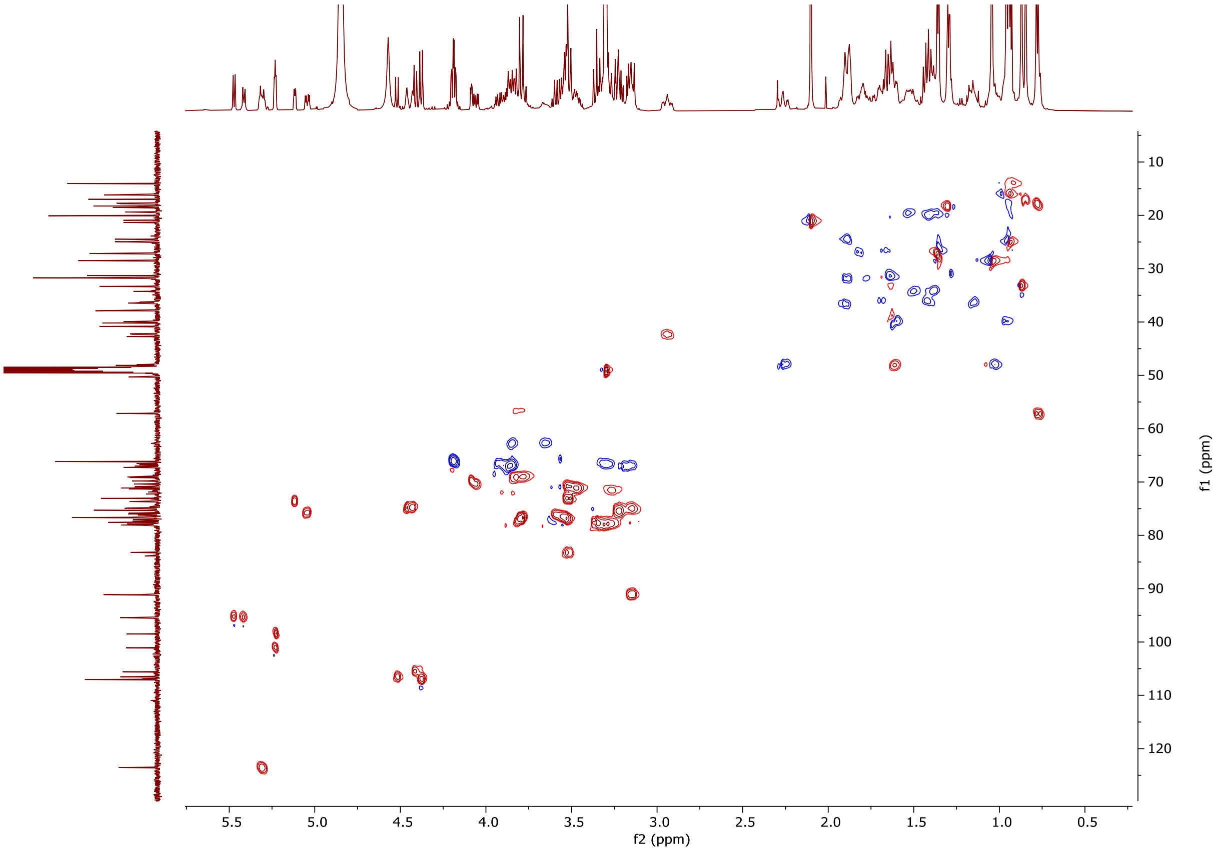

Figure S21. HSQC spectrum of compound **2/3**

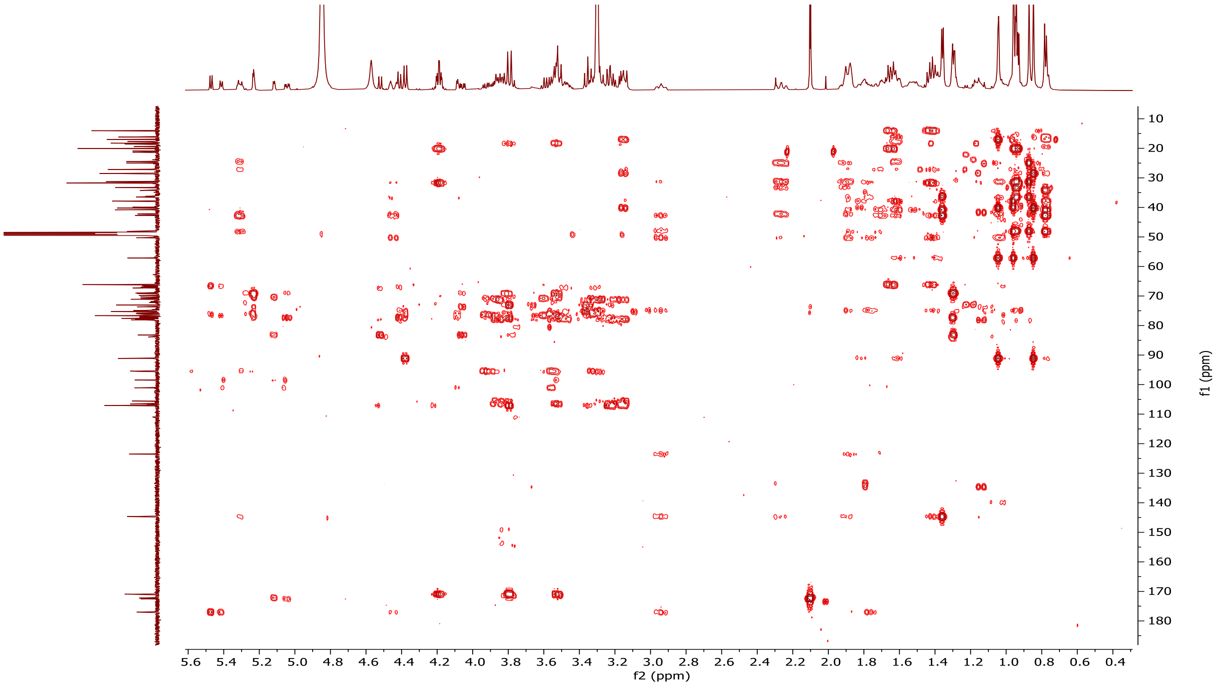

Figure S22. HMBC spectrum of compound **2/3**

HEJ-MASS LAB -ICCBS | JEOL HX 110 MASS SPECTROMETER (FAB-HR)

Measured MASS	Theoretical MASS	Delta	Delta	RDB	Composition
1099.5355	1099.536	-0.5	-0.5	35.5	C72 H75 O10
	1099.5325	2.7	3	13.5	C54 H83 O23
	1099.5266	8.1	8.9	22.5	C61 H79 O18
	1099.5478	-11.2	-12.3	17.5	C58 H83 O20

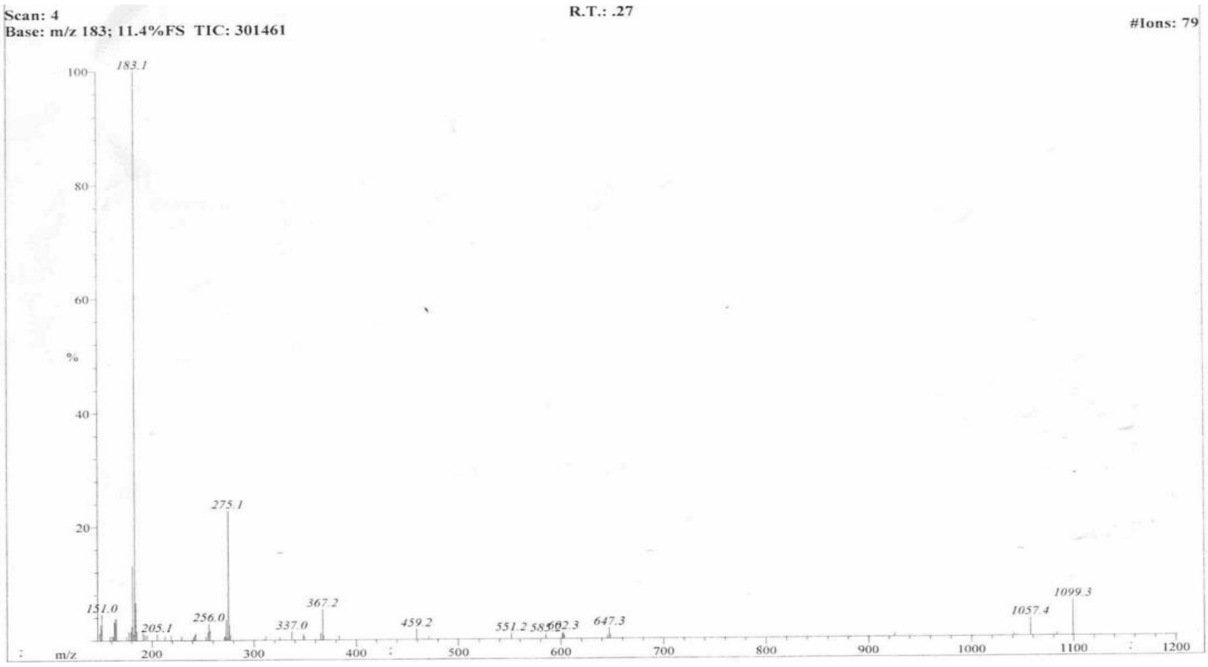

Figure S23. HRFABMS spectrum of compound **4/5**

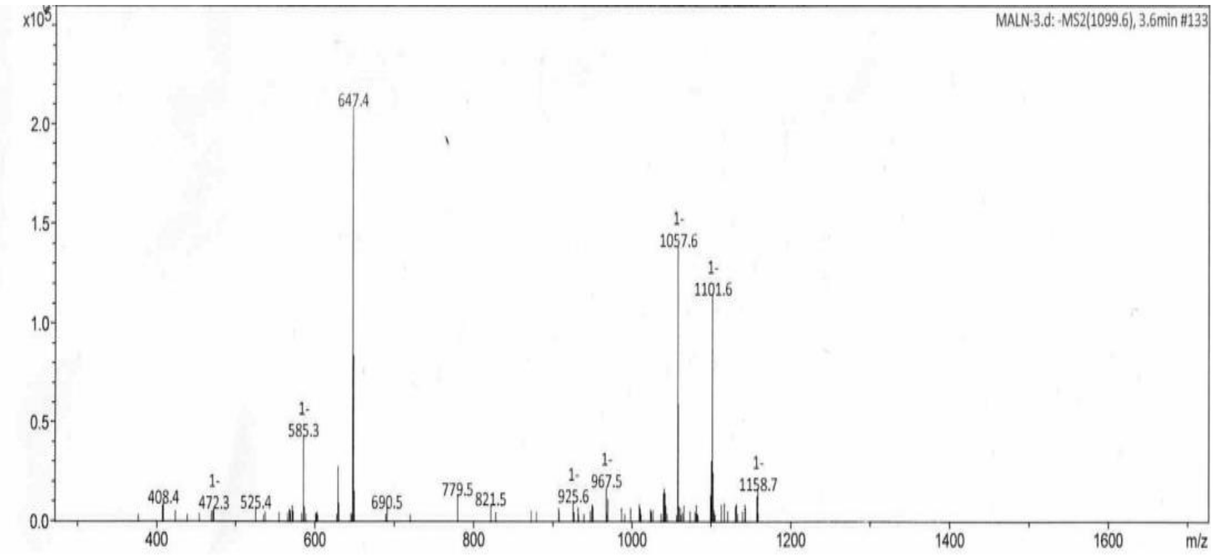

Figure S24. ESI-MS^2 spectrum of *m/z* = 1099.6 of compound **4/5**

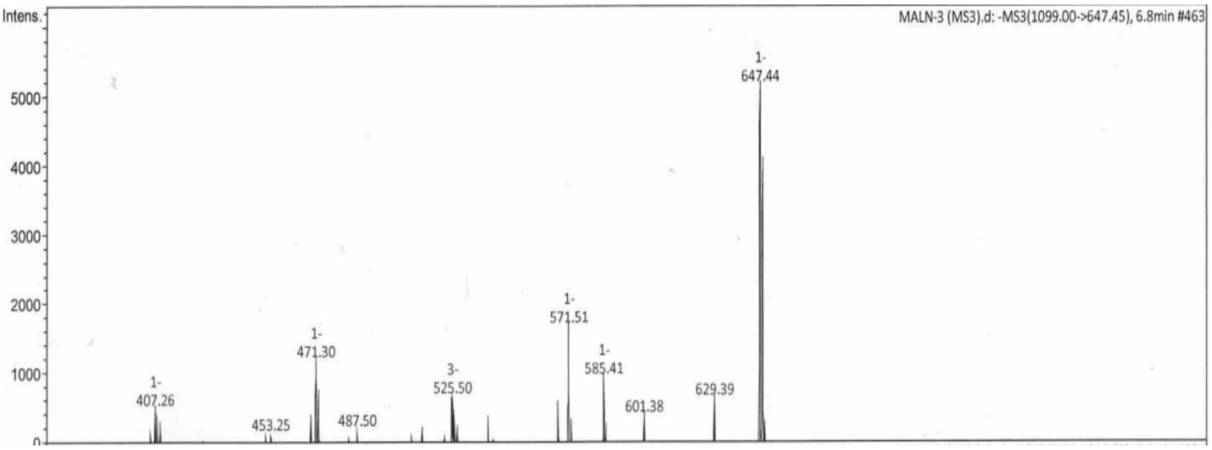

Figure S25. ESI-MS^3 spectrum of *m/z* = 647.4 of compound **4/5**

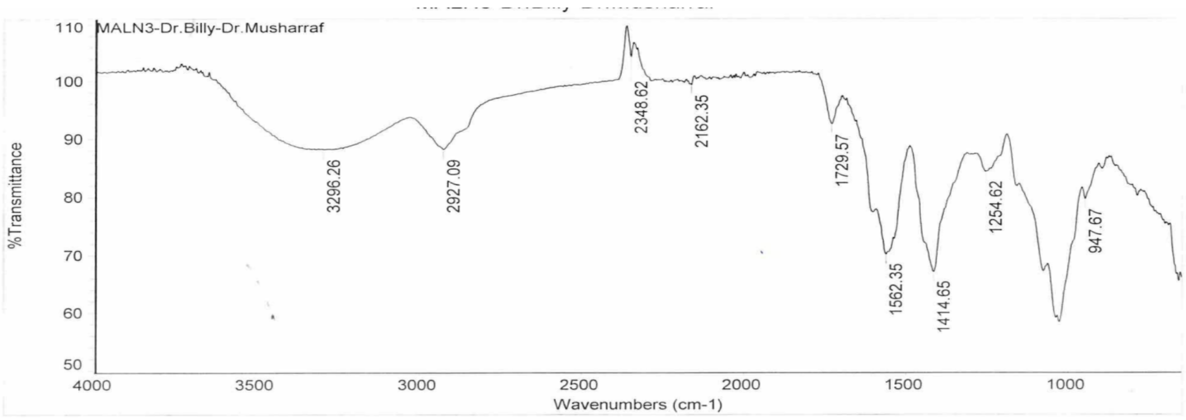

Figure S26. IR spectrum of compound **4/5**

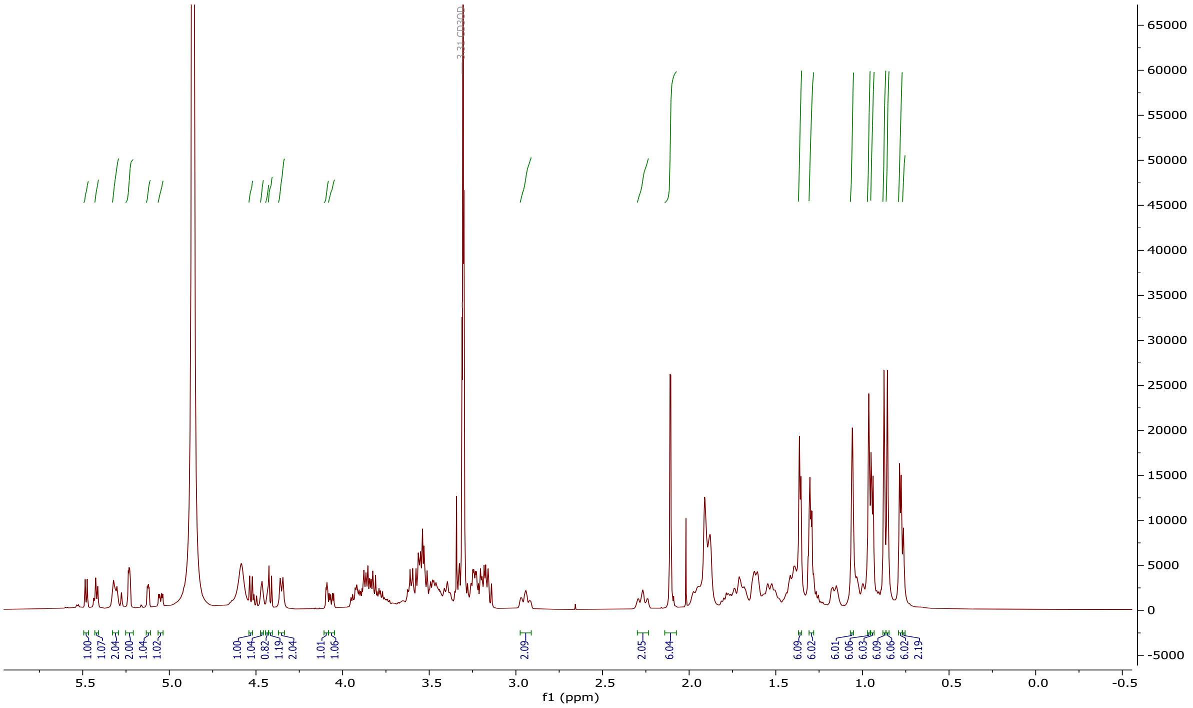

Figure S27. ^{1}H NMR (600 MHz, CD_3OD) spectrum of compound **4/5**

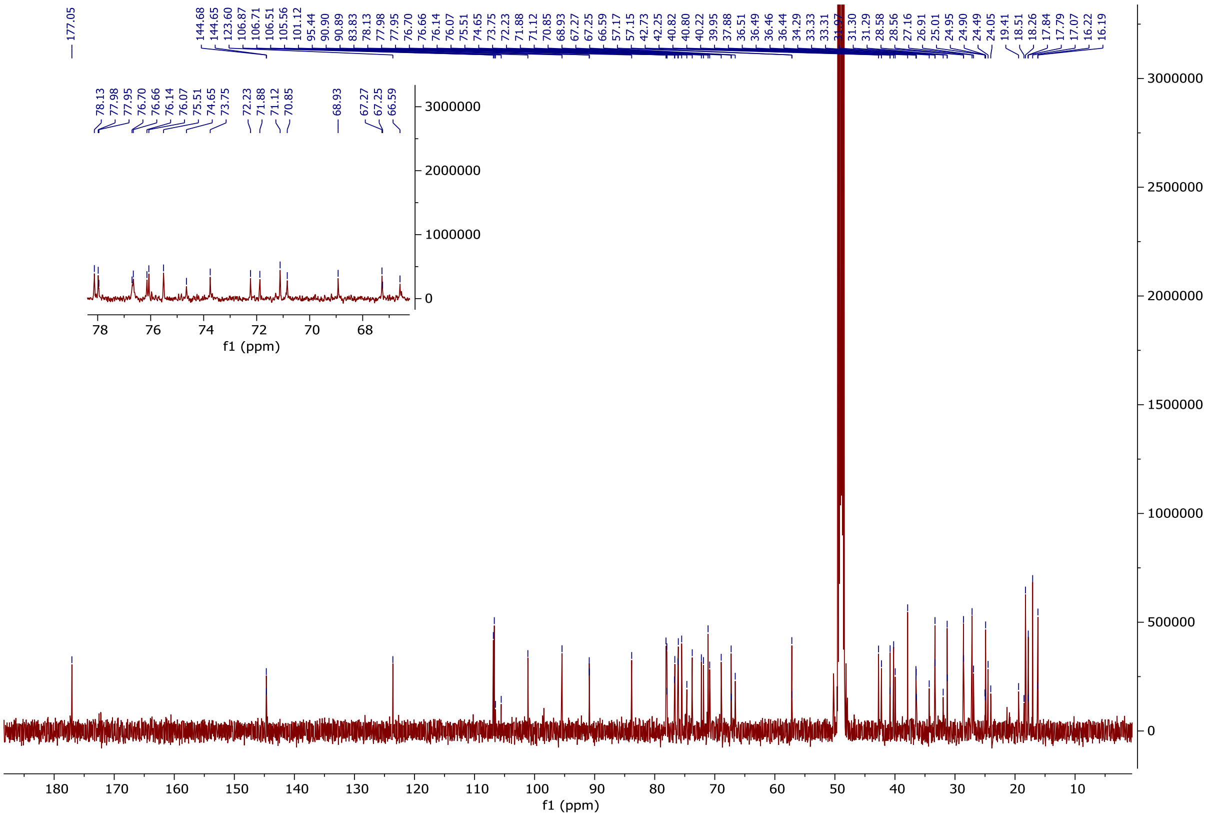

Figure S28. ^{13}C NMR (150 MHz, CD_3OD) spectrum of compound **4/5**

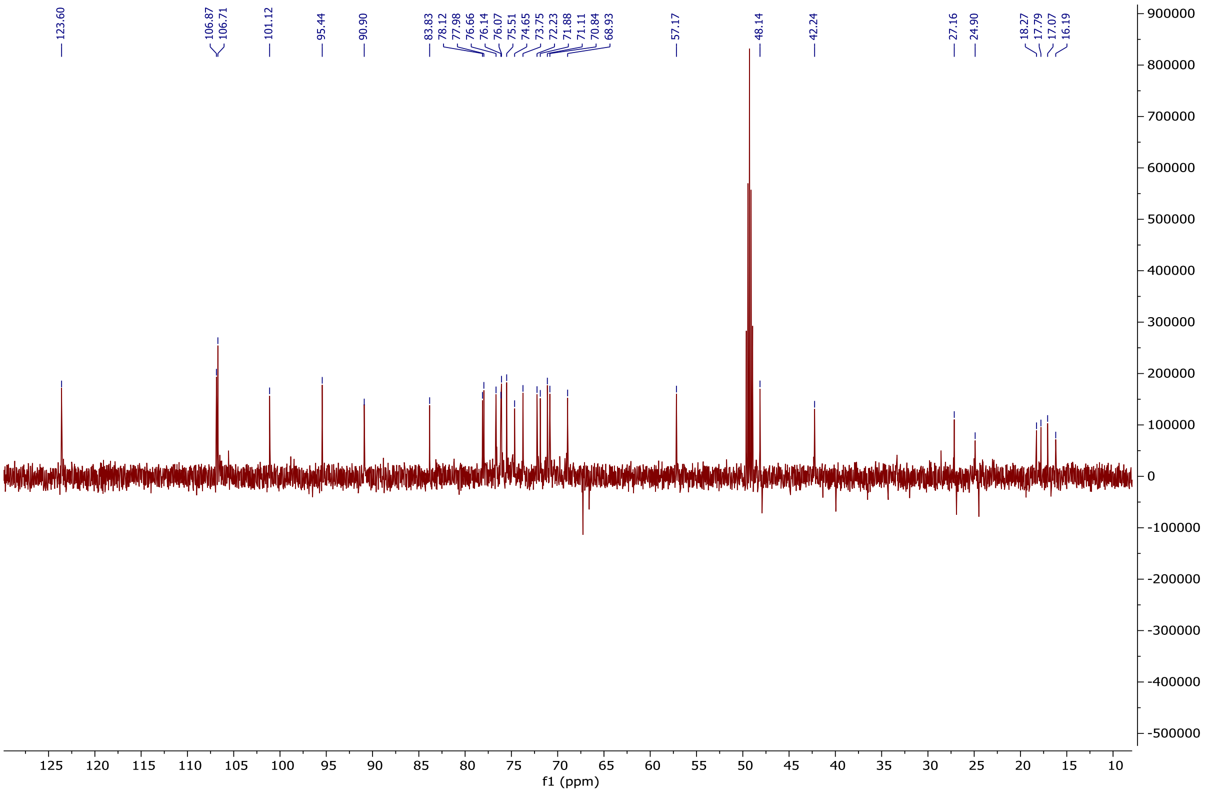

Figure S29. DEPT 135 spectrum of compound **4/5**

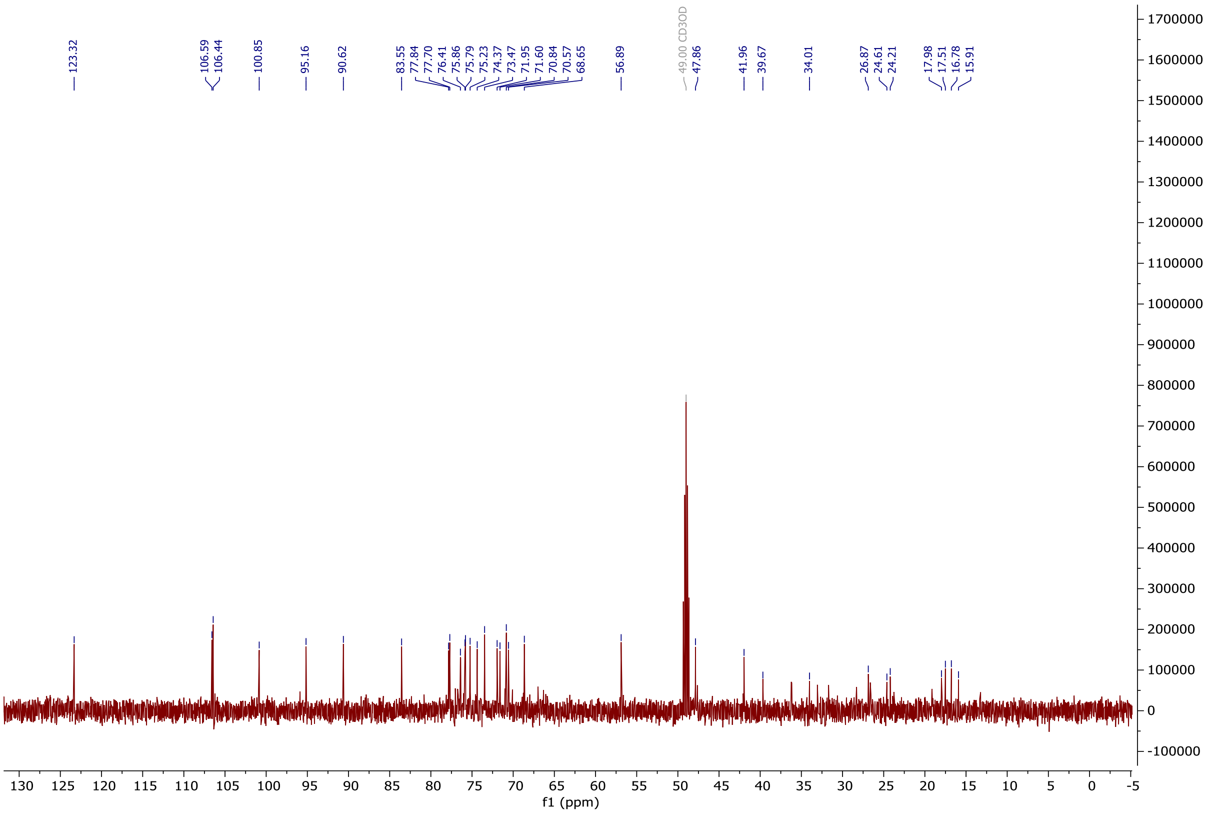

Figure S30. DEPT 90 spectrum of compound **4/5**

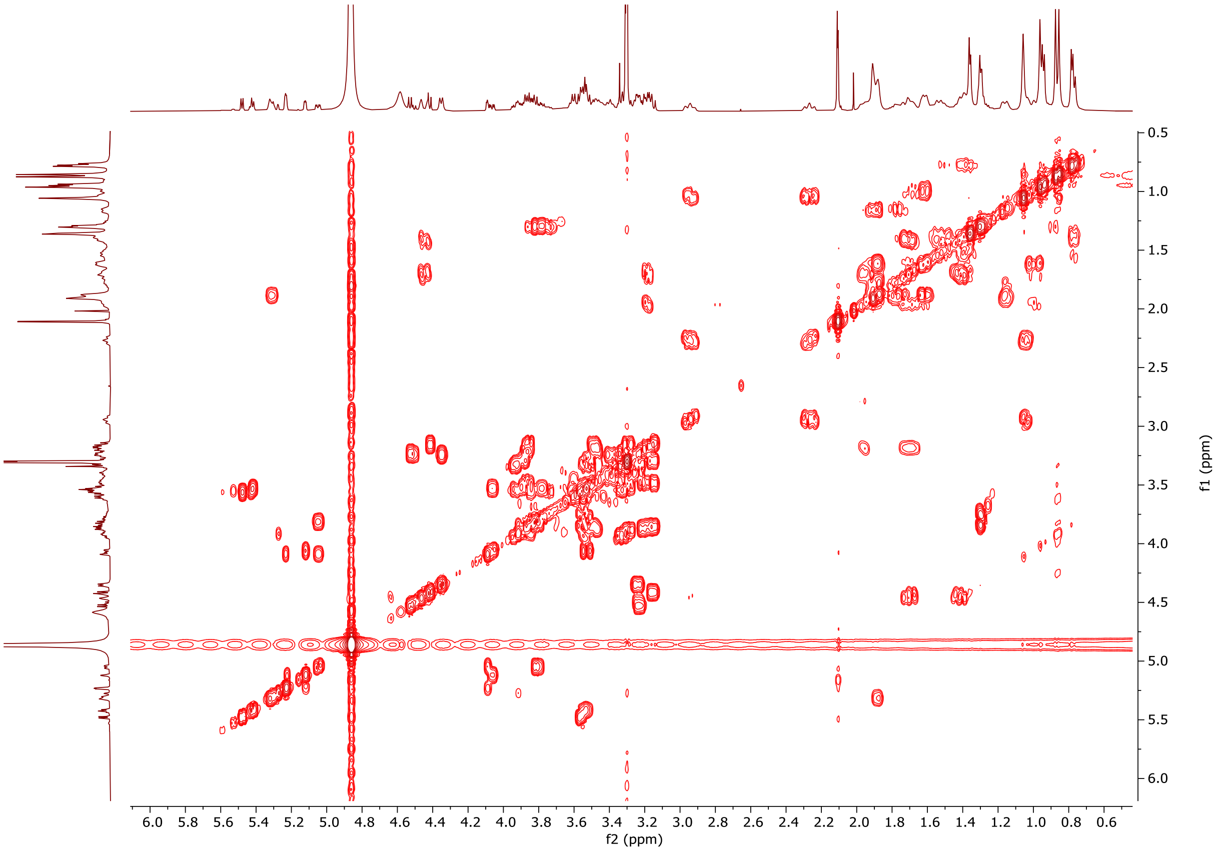

Figure S31. ^{1}H–^{1}H COSY spectrum of compound **4/5**

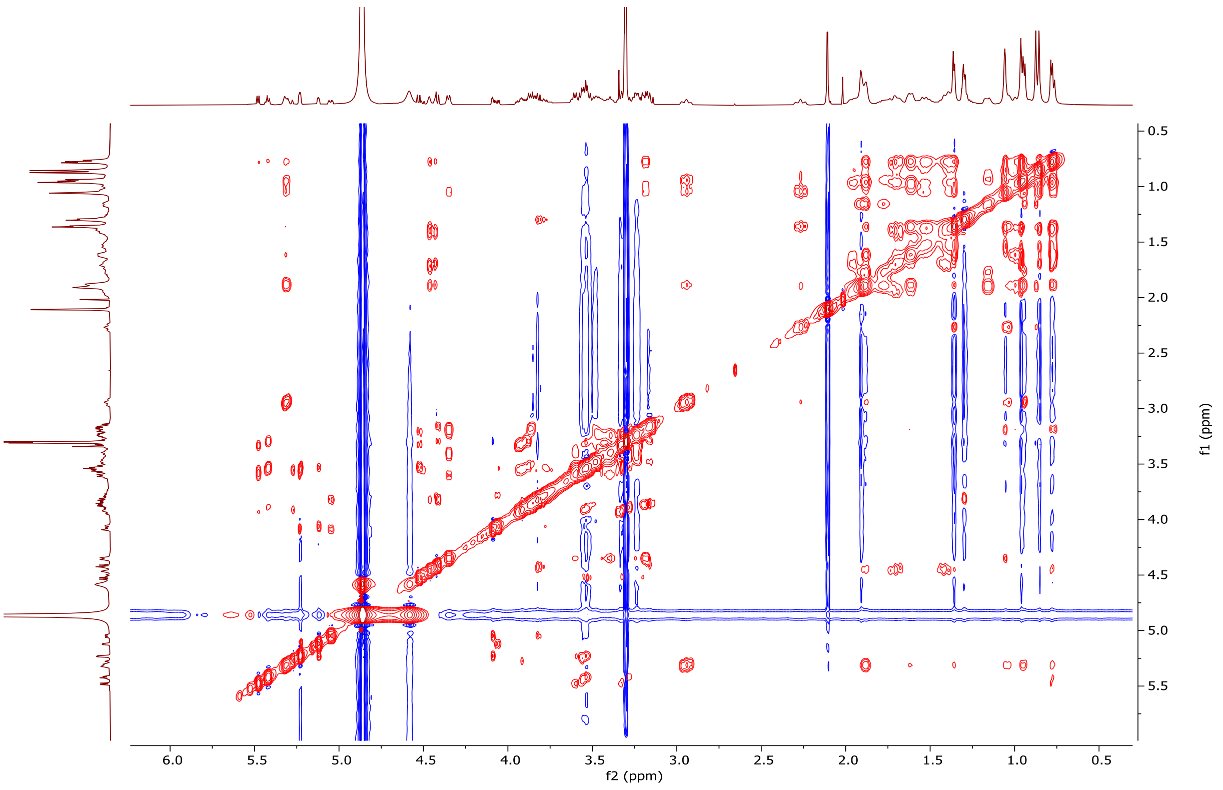

Figure S32. NOESY spectrum of compound **4/5**

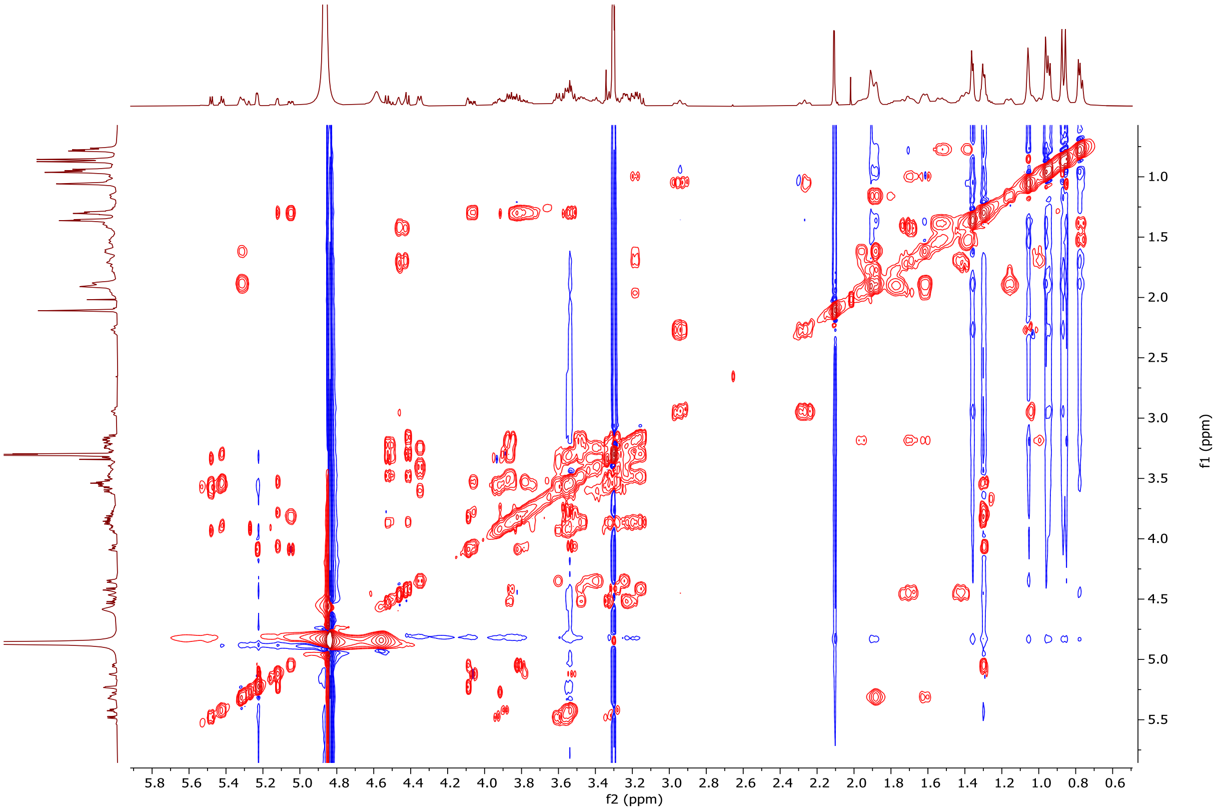

Figure S33. TOCSY spectrum of compound **4/5**

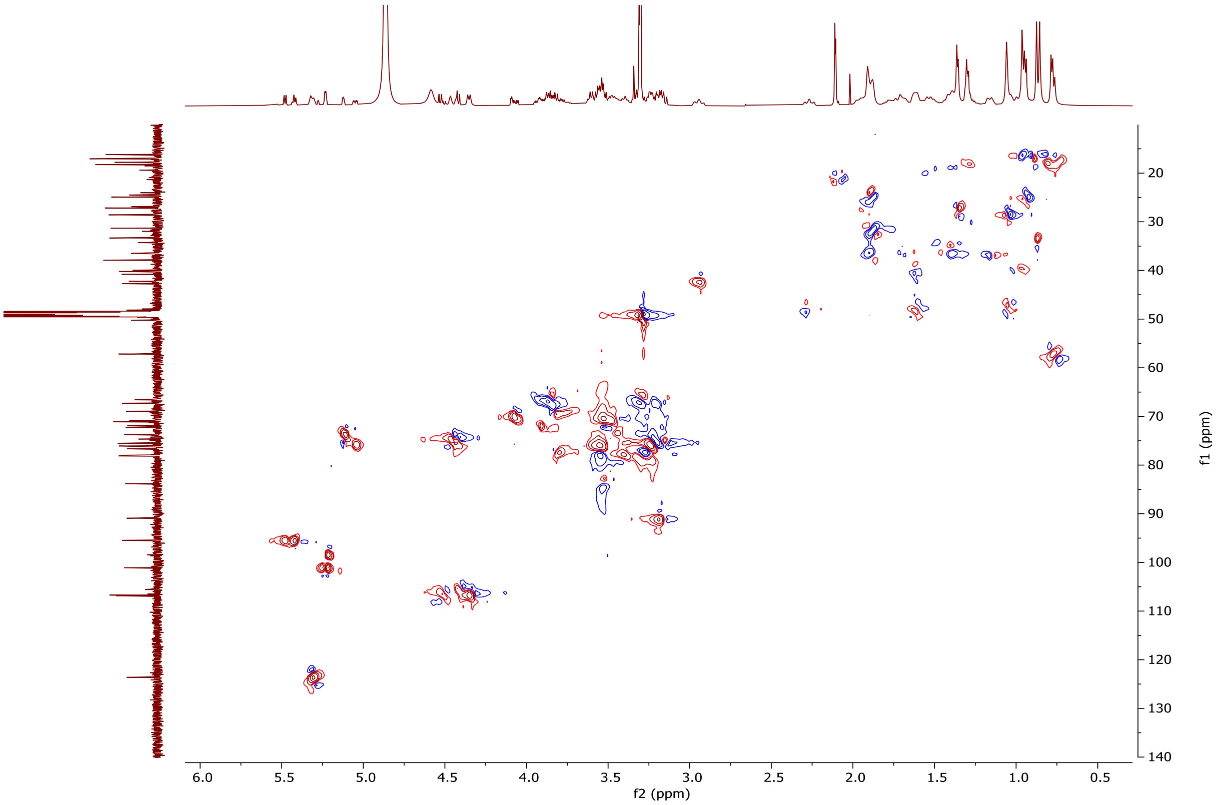

Figure S34. HSQC spectrum of compound **4/5**

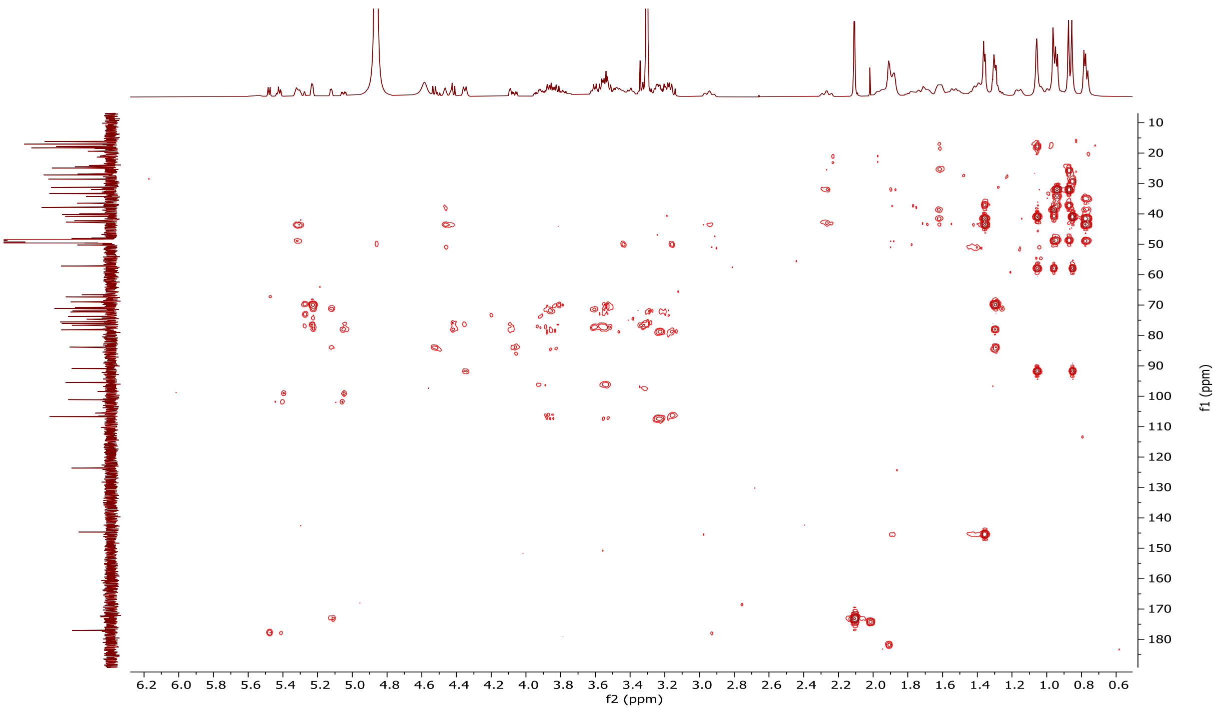

Figure S35. HMBC spectrum of compound **4/5**

HEJ-MASS LAB -ICCBS | JEOL HX 110 MASS SPECTROMETER (FAB-HR)

Measured MASS	Theoretical MASS	Delta [ppm]	Delta [mmu]	RDB	Composition
967.4951	967.4938	1.4	1.3	34.5	C67 H67 O6
	967.4996	-4.7	-4.5	25.5	C60 H71 O11
	967.4903	5	4.8	12.5	C49 H75 O19
	967.5055	-10.8	-10.4	16.5	C53 H75 O16
	967.4844	11.1	10.7	21.5	C56 H71 O14
	967.509	-14.4	-13.9	38.5	C71 H67 O3

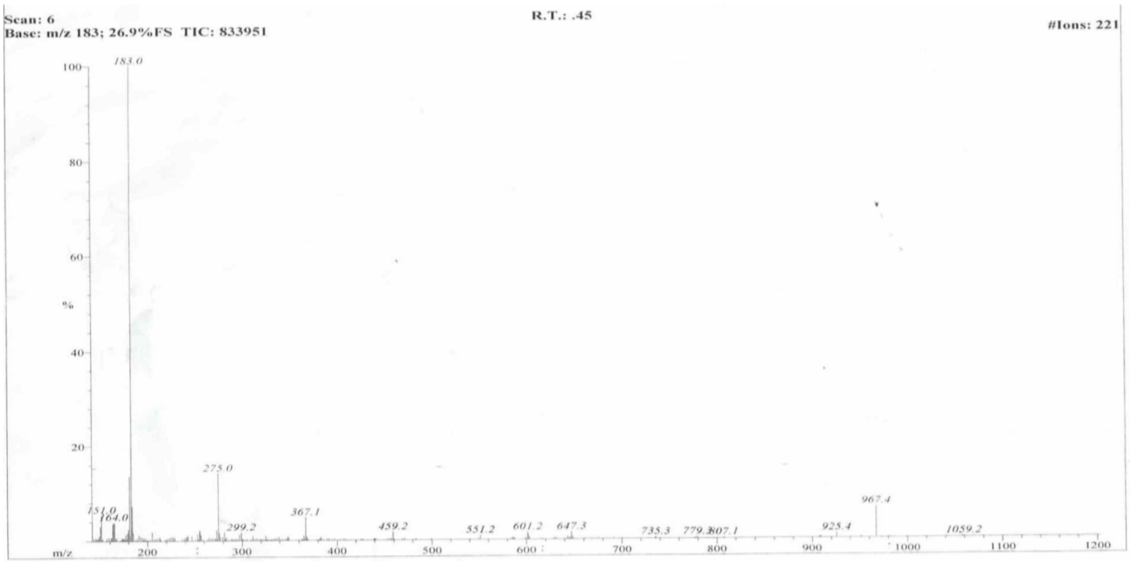

Figure S36. HRFABMS spectrum of compounds **6−8**

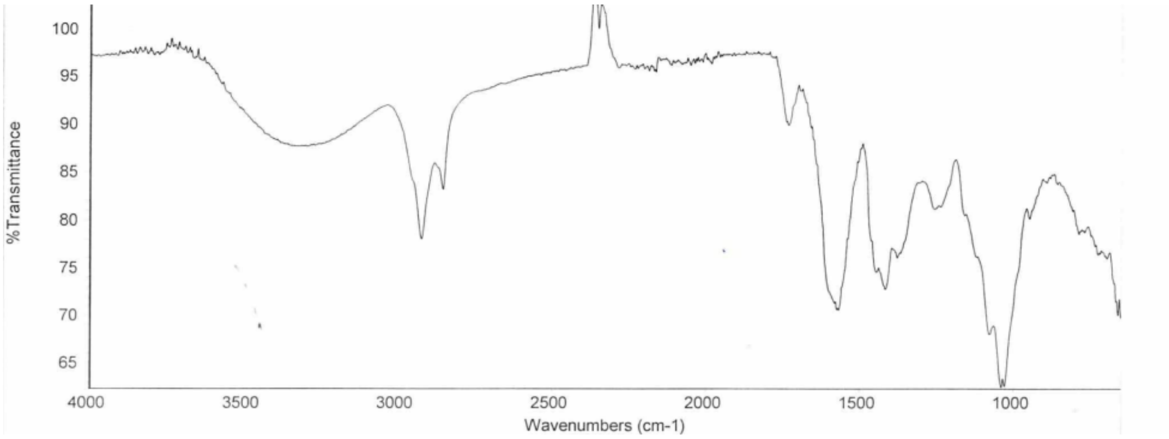

Figure S37. IR spectrum of compound **6−8**

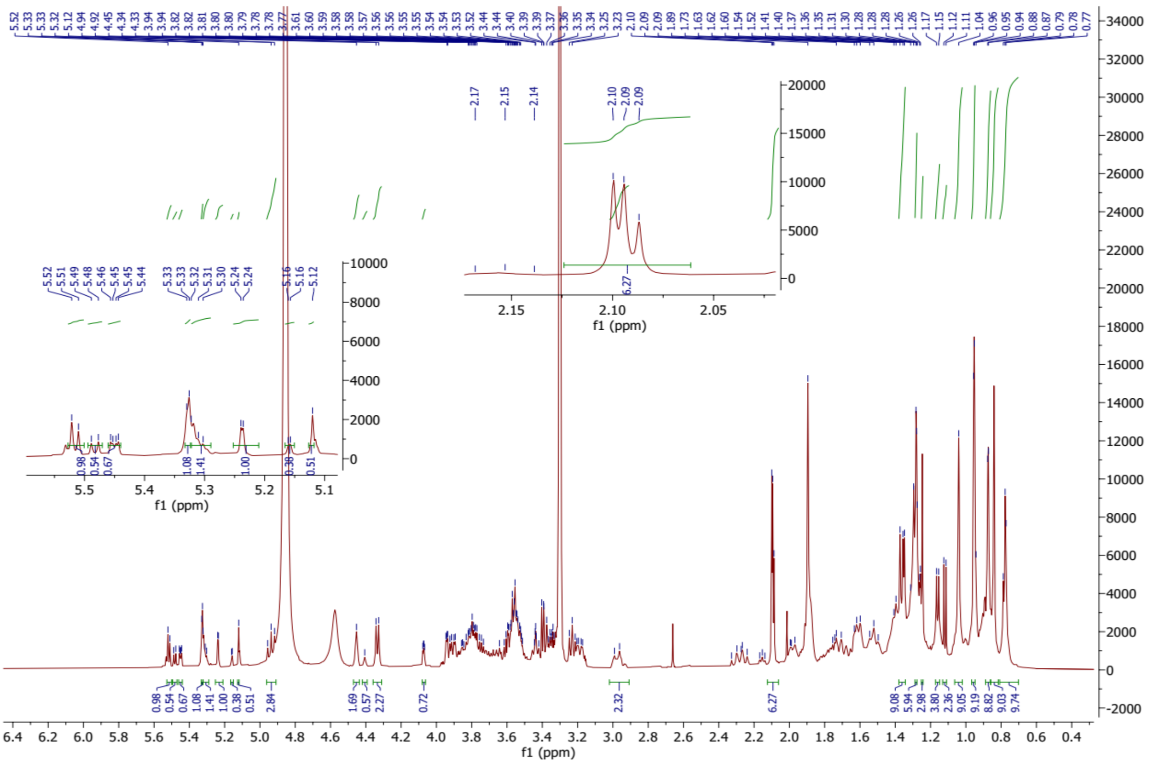

Figure S38. ^{1}H NMR (800 MHz, CD_3OD) spectrum of compound **6−8**

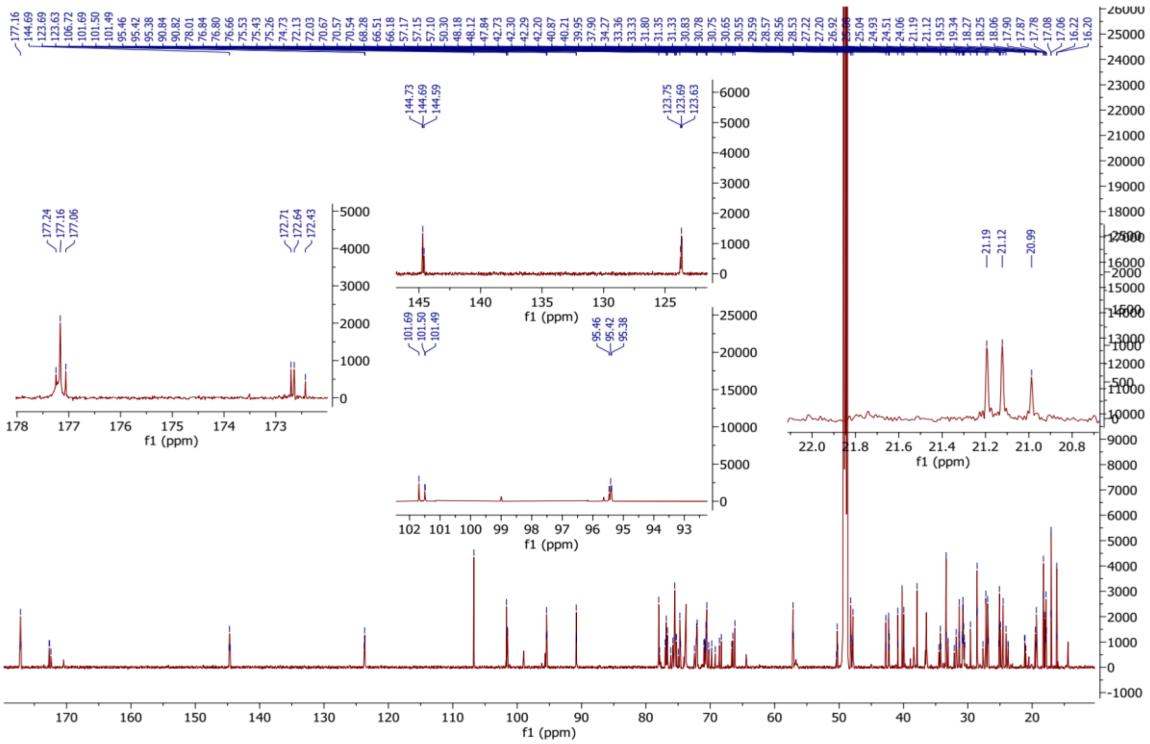

Figure S39. ^{1}H NMR (200 MHz, CD_3OD) spectrum of compound **6−8**

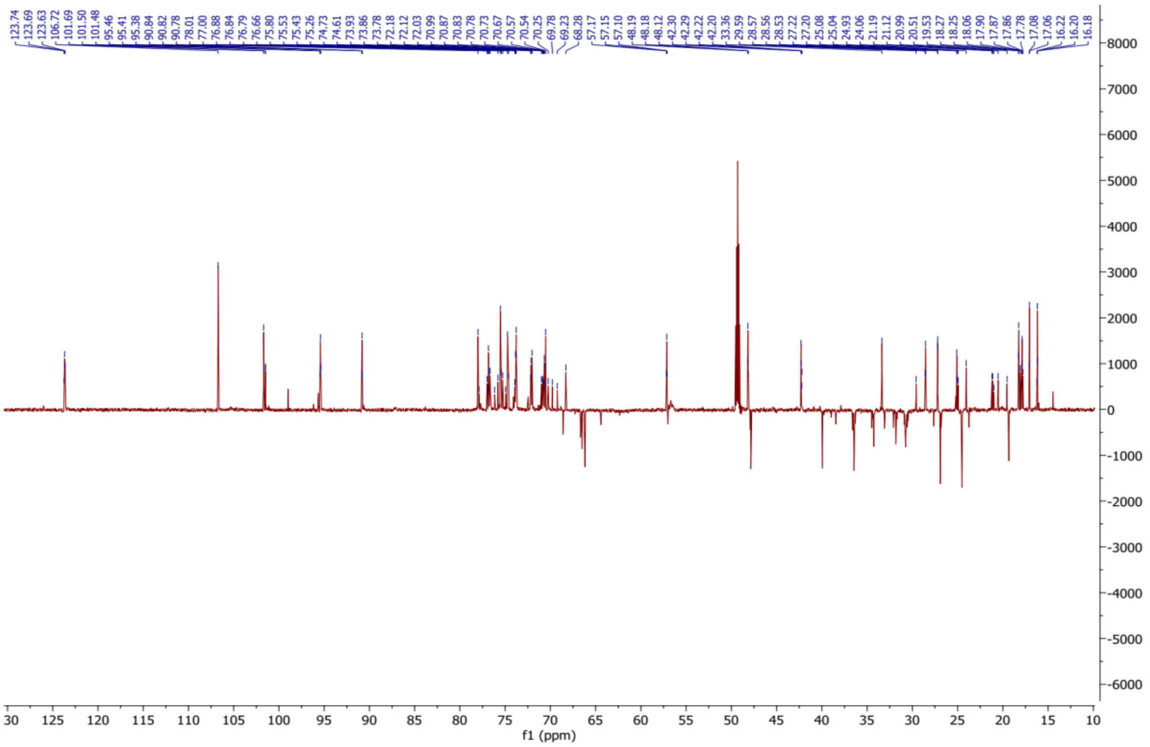

Figure S40. DEPT 135 spectrum of compound **6−8**

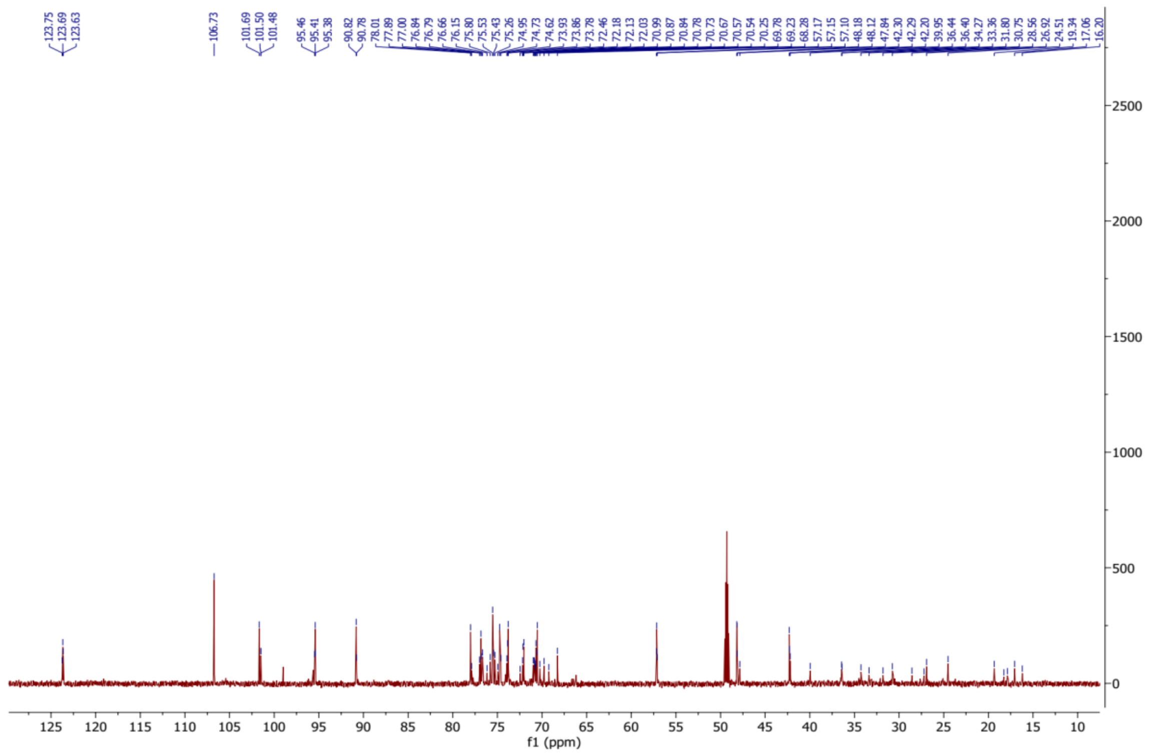

Figure S41. DEPT 90 spectrum of compound **6−8**

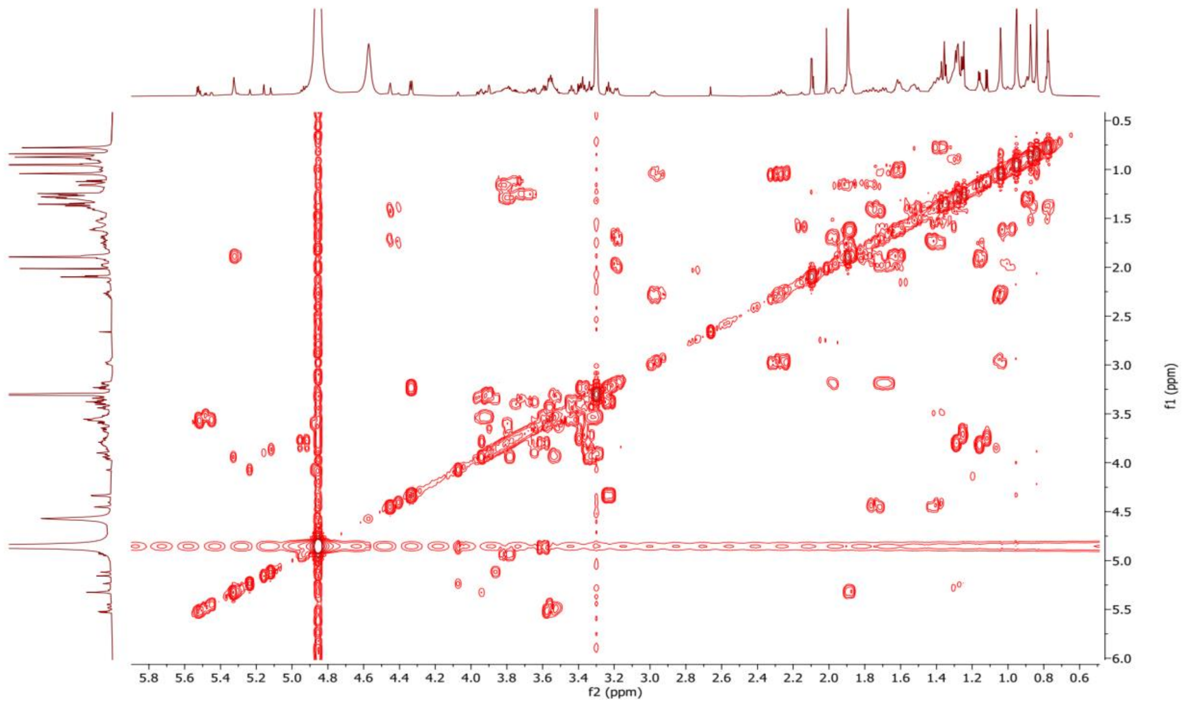

Figure S42. ^{1}H–^{1}H COSY spectrum of compound **6−8**

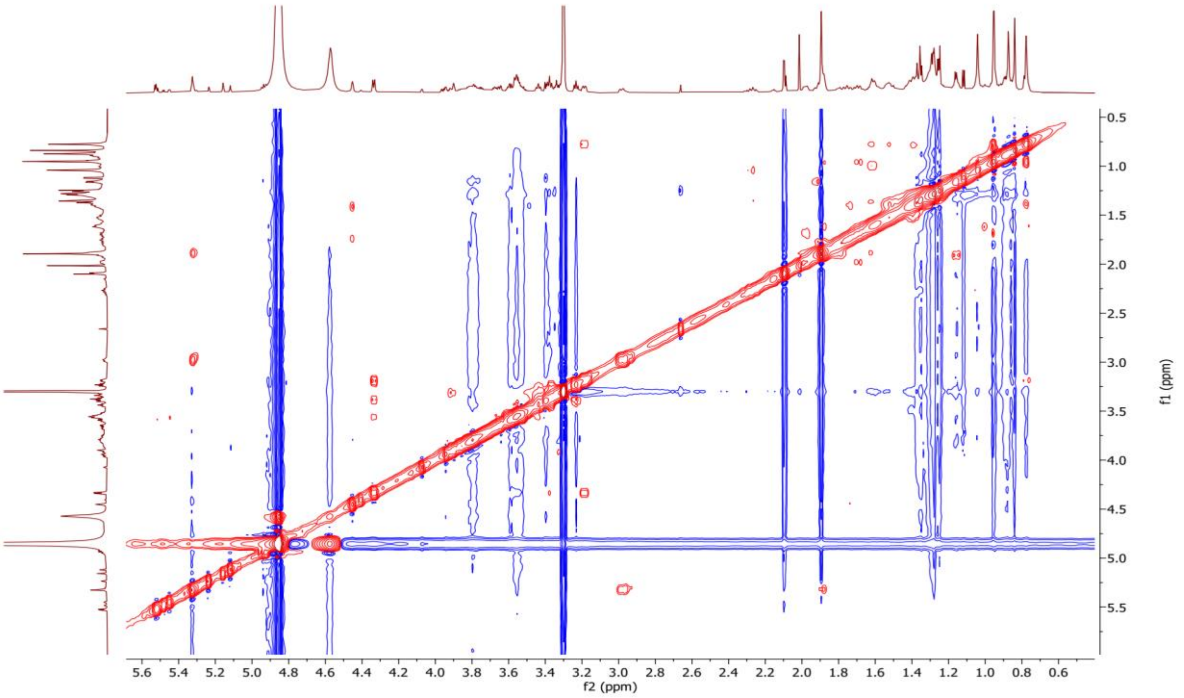

Figure S43. NOESY spectrum of compound **6−8**

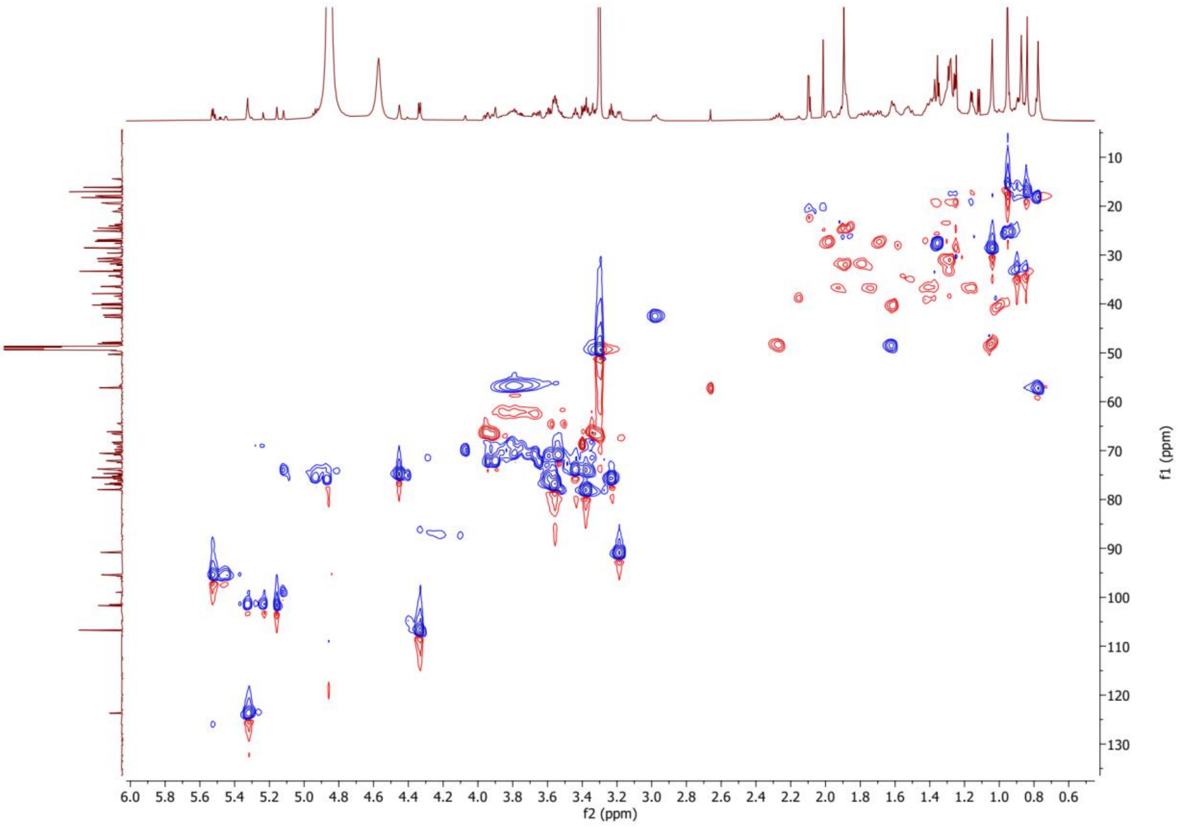

Figure S44. HSQC spectrum of compound **6−8**

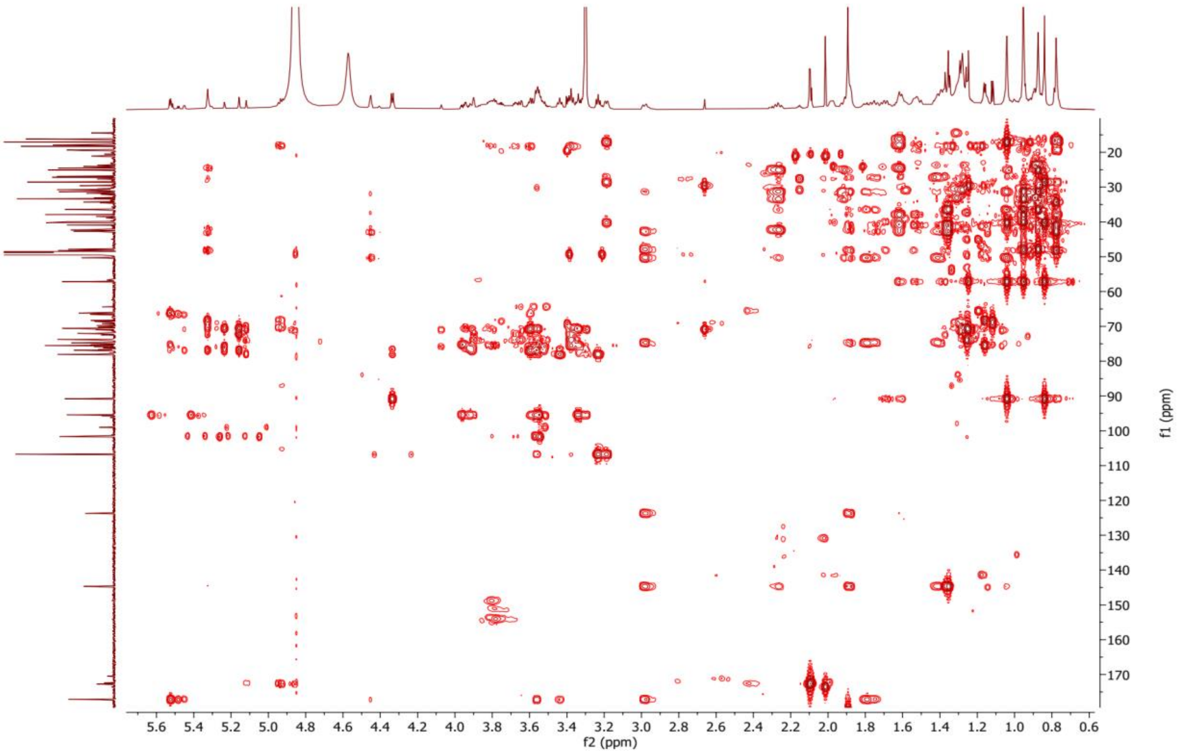

Figure S45. HBMC spectrum of compound **6−8**

HEJ-MASS LAB -ICCBS

JEOL HX 110 MASS SPECTROMETER (FAB-HR)

Measured MASS	Theoretical MASS	Delta	Delta	RDB	Composition
1113.5881	1113.5845	3.2	3.6	12.5	C56 H89 O22
	1113.5787	8.5	9.4	21.5	C63 H85 O17
	1113.5998	-10.5	-11.7	16.5	C60 H89 O19
	1113.6057	-15.8	-17.6	7.5	C53 H93 O24
	1113.5693	16.9	18.8	8.5	C52 H89 O25

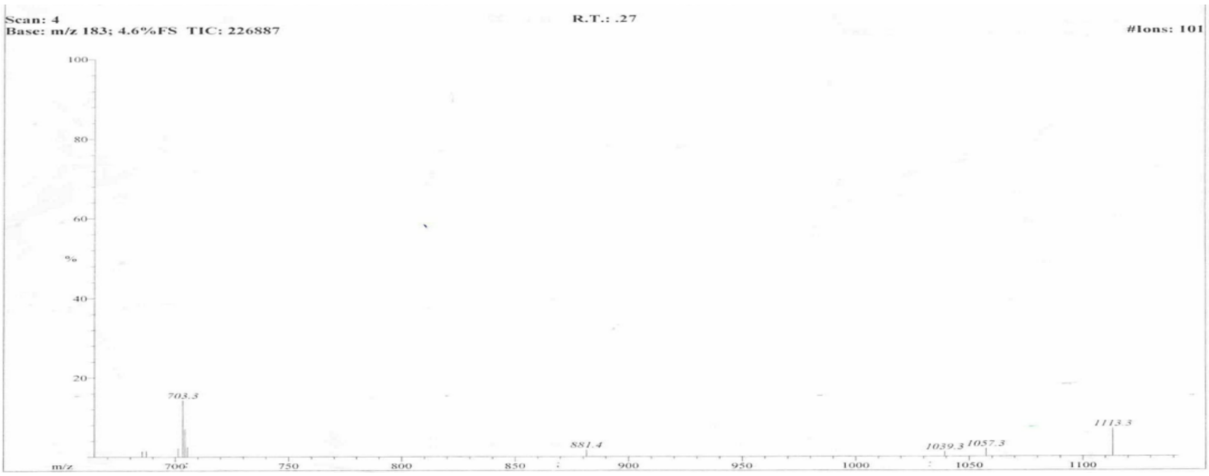

Figure S46. HRFABMS spectrum of compound **9**

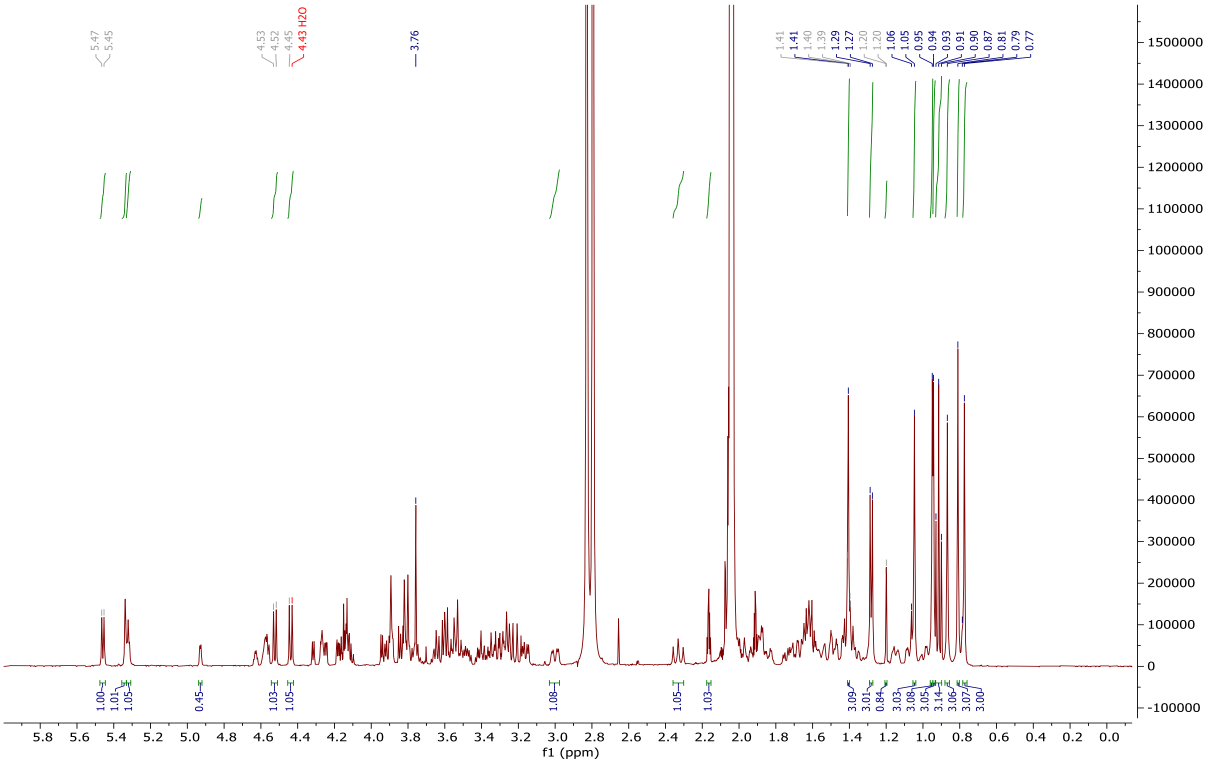

Figure S47. ^{1}H NMR (600 MHz, CD_3OD) spectrum of compound **9**

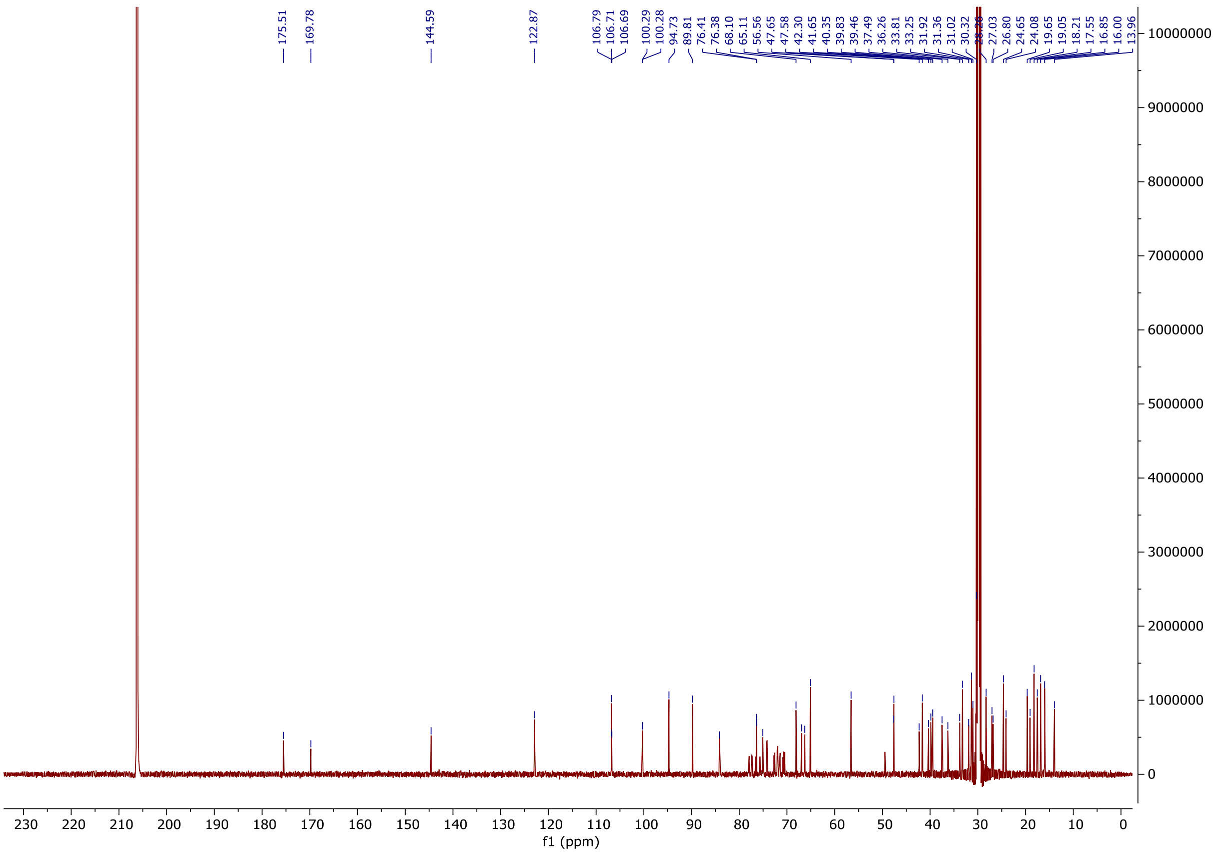

Figure S48. ^{13}C NMR (150 MHz, CD_3OD) spectrum of compound **9**

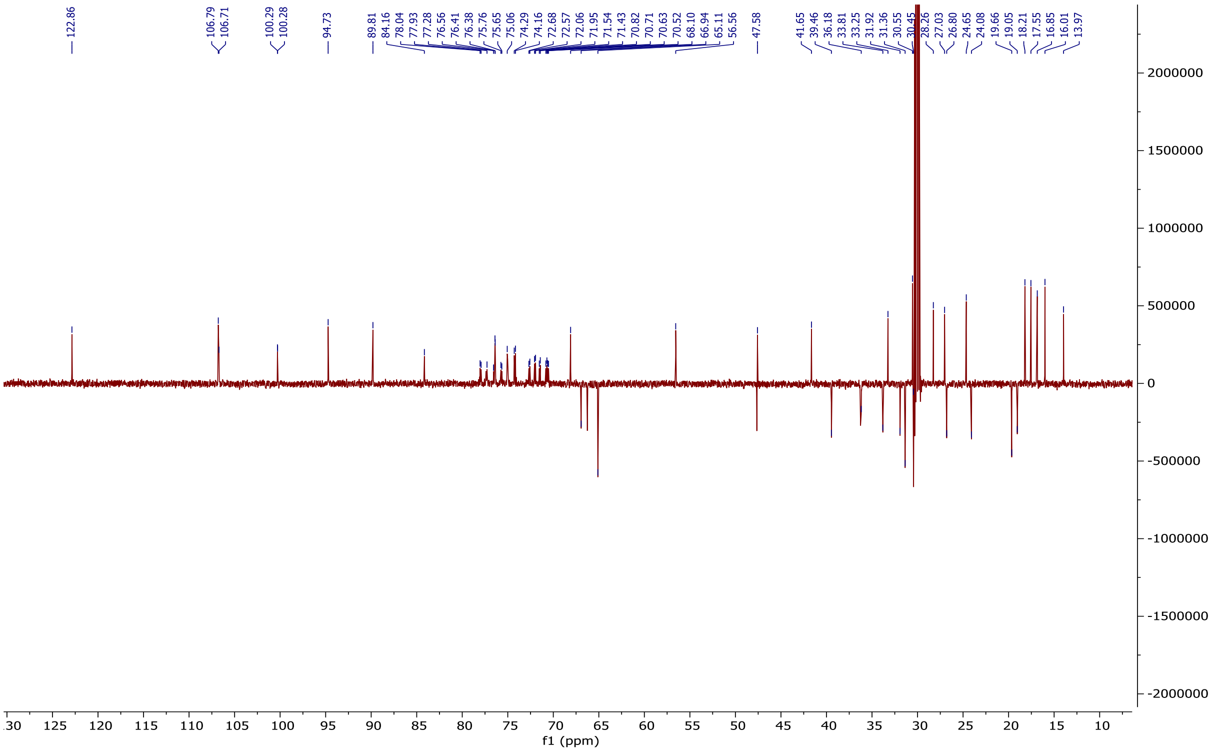

Figure S49. DEPT 135 spectrum of compound **9**

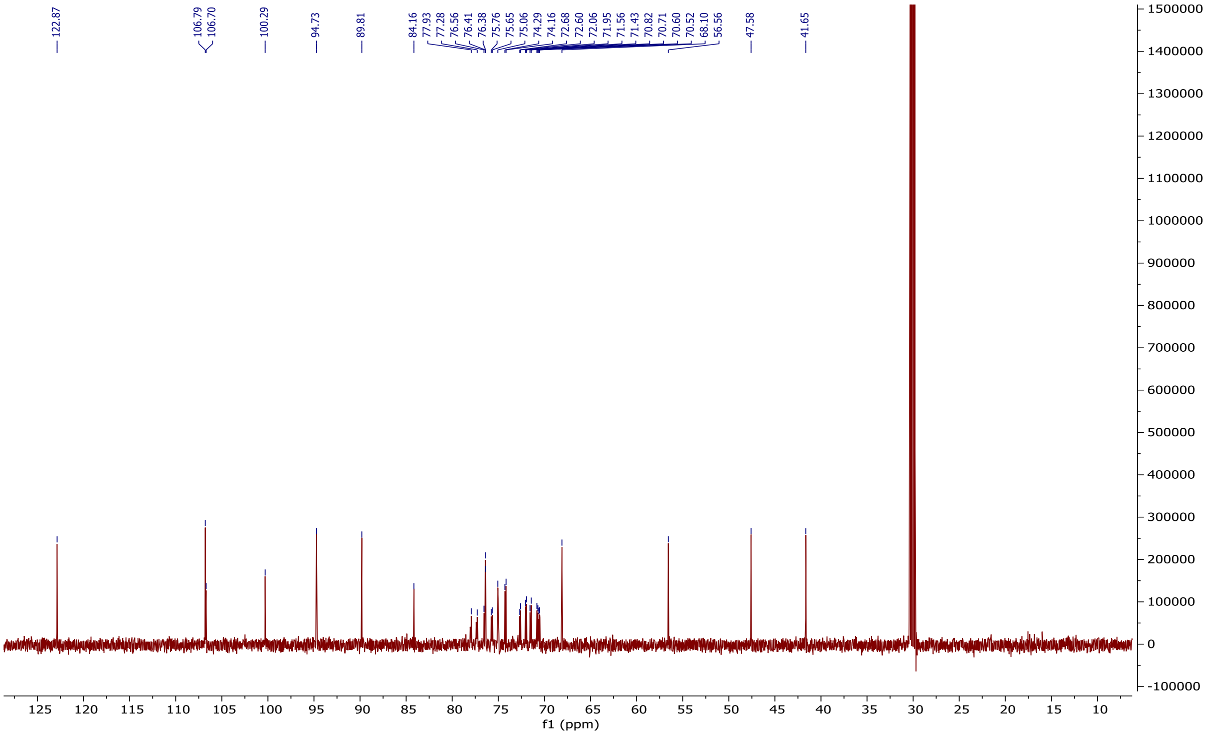

Figure S50. DEPT 90 spectrum of compound **9**

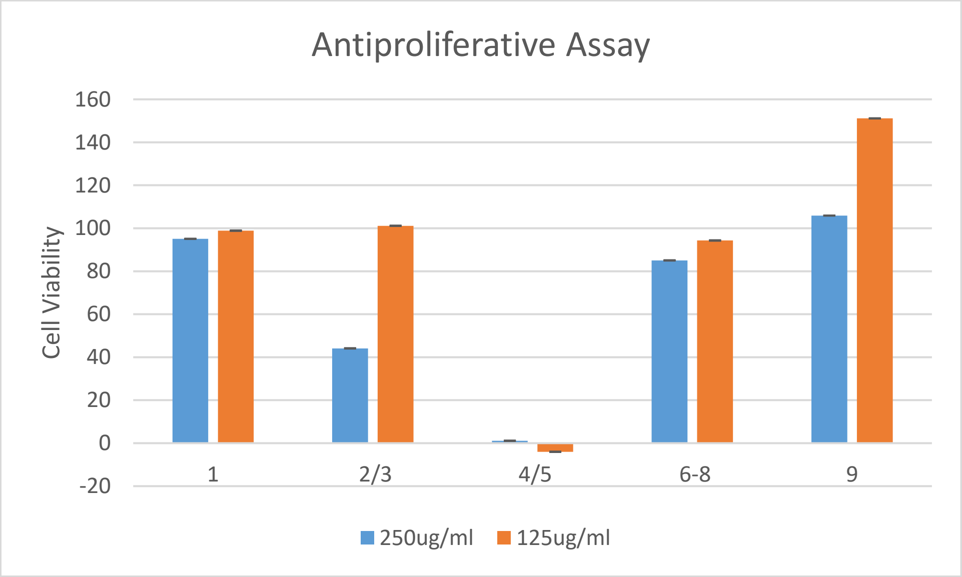

Figure S51. Cell viability of all tested compounds.
